# Combining OSMAC, metabolomic and genomic methods for the production and annotation of halogenated azaphilones and ilicicolins in termite symbiotic fungi

**DOI:** 10.1038/s41598-022-22256-3

**Published:** 2022-10-15

**Authors:** Téo Hebra, Nicolas Pollet, David Touboul, Véronique Eparvier

**Affiliations:** 1 Université Paris-Saclay, CNRS, Institut de Chimie Des Substances Naturelles, UPR 2301, 91198 Gif-Sur-Yvette, France; 2 Université Paris-Saclay, CNRS, IRD, Évolution Génomes Comportement & Écologie, 91198 Gif-Sur-Yvette, France; 3 Laboratoire de Chimie Moléculaire (LCM), CNRS UMR 9168, École Polytechnique, Institut Polytechnique de Paris, 91128 Palaiseau, France

**Keywords:** Metabolic pathways, Metabolomics, Natural products, Small molecules

## Abstract

We gathered a collection of termite mutualistic strains from French Guiana to explore the metabolites of symbiotic microorganisms. Molecular networks reconstructed from a metabolomic analysis using LC–ESI–MS/MS methodology led us to identify two families of chlorinated polyketides, i.e.*,* azaphilones from *Penicillium sclerotiorum* and ilicicolins from *Neonectria discophora*. To define the biosynthetic pathways related to these two types of scaffolds, we used a whole genome sequencing approach followed by hybrid assembly from short and long reads. We found two biosynthetic gene clusters, including two FAD-dependent halogenases. To exploit the enzymatic promiscuity of the two identified FAD halogenases, we sought to biosynthesize novel halogenated metabolites. An OSMAC strategy was used and resulted in the production of brominated analogs of ilicicolins and azaphilones as well as iodinated analogs of azaphilones.

## Introduction

Natural products have been sources of new active molecules for several decades and have been exploited in pharmacology, agrochemistry and cosmetology. One major challenge in natural product research is the ability to identify new compounds with biological activity. For this purpose, it is possible to screen extract libraries to isolate new molecules^1^, to diversify the chemical moiety of a natural product skeleton by hemisynthesis^2^, or to identify and use enzymes as biocatalysts^3^. An alternative method for discovering new biosynthesized molecular scaffolds is to explore unusual ecological niches, such as insect-associated microorganisms, which are the subject of growing interest. Indeed, we can explore the chemosphere of the one million five thousand insect species that have been formally described^4^. Insects colonize almost all terrestrial habitats, including forests, deserts, and coasts. Moreover, these insects host numerous symbiotic microorganisms in different organs, such as the cuticle, digestive system, and glands^5^. Many insect-microorganism interactions have been studied within eusocial insects named Apocrites (e.g., bees, wasps, ants), but few studies have been performed on termite-microorganism associations outside trophobiosis^6–13^.

We previously described examples of antimicrobial compounds produced by termite symbiotic/associated microorganisms or microorganisms co-occuring with termites^6,14,20^. In particular, we isolated chlorinated metabolites of the ilicicolin and azaphilone families from *Neonectria discophora* and *Penicillium sclerotiorum* fungal strains^18–20^. In microorganisms, these halogenated compounds have been mainly isolated from fungi in marine environments^21^. In recent decades, their pharmaceutical and medical applications have been explored, and biological properties such as anticancer, antiviral, antibacterial, anti-inflammatory, antifungal, antifouling and insecticidal activity have been reported^22^. As an example, halogenated azaphilones are reported as antimicrobial compounds but also natural pigments^23,24^, and ilicicolins are reported as anti-trypanosomes or antimicrobials^25^.

Dereplication strategies based on metabolomic approaches have increased the possibilities of identifying novel chemical scaffolds or novel analogs of molecules in complex extracts^26^. In particular, tandem mass spectrometry (MS/MS) data can be clustered using molecular networks to identify structural similarities of related molecules^27^. Although halogenated compounds can be annotated using such approaches, understanding their biosynthetic pathways is crucial to deeply explore molecular diversity^28,29^. These biosynthetic pathways can now be identified from complete microorganism genomes assembled using next-generation sequencing methods^30^. Biosynthetic clusters of specialized metabolites in genomes can be automatically identified using computational approaches such as antiSMASH^31^. The production of novel compounds can then be conceived and produced by exploiting enzymatic promiscuity^32,33^. Finally, to generate greater chemical diversity and produce cryptic specialized metabolites, the One Strain Many Compounds (OSMAC) method^34–37^ can be employed to increase the chemodiversity of halogenated compounds.

The objective of this study was to combine genomic and metabolomic approaches to study two families of halogenated polyketides produced by mutualistic fungal strains of termites from French Guiana, i.e., ilicicolines and azaphilones. We annotated the chemodiversity of halogenated compounds and deciphered their biosynthetic pathways. In this study, the metabolism of the two model strains was also controlled to produce original analogs of ilicicolin and azaphilone.

## Results and discussion

### Dereplication of *Neonectria discophora* SNB-CN63 metabolomes and *Penicillium sclerotiorum* SNB-CN111

**Figure 1:**
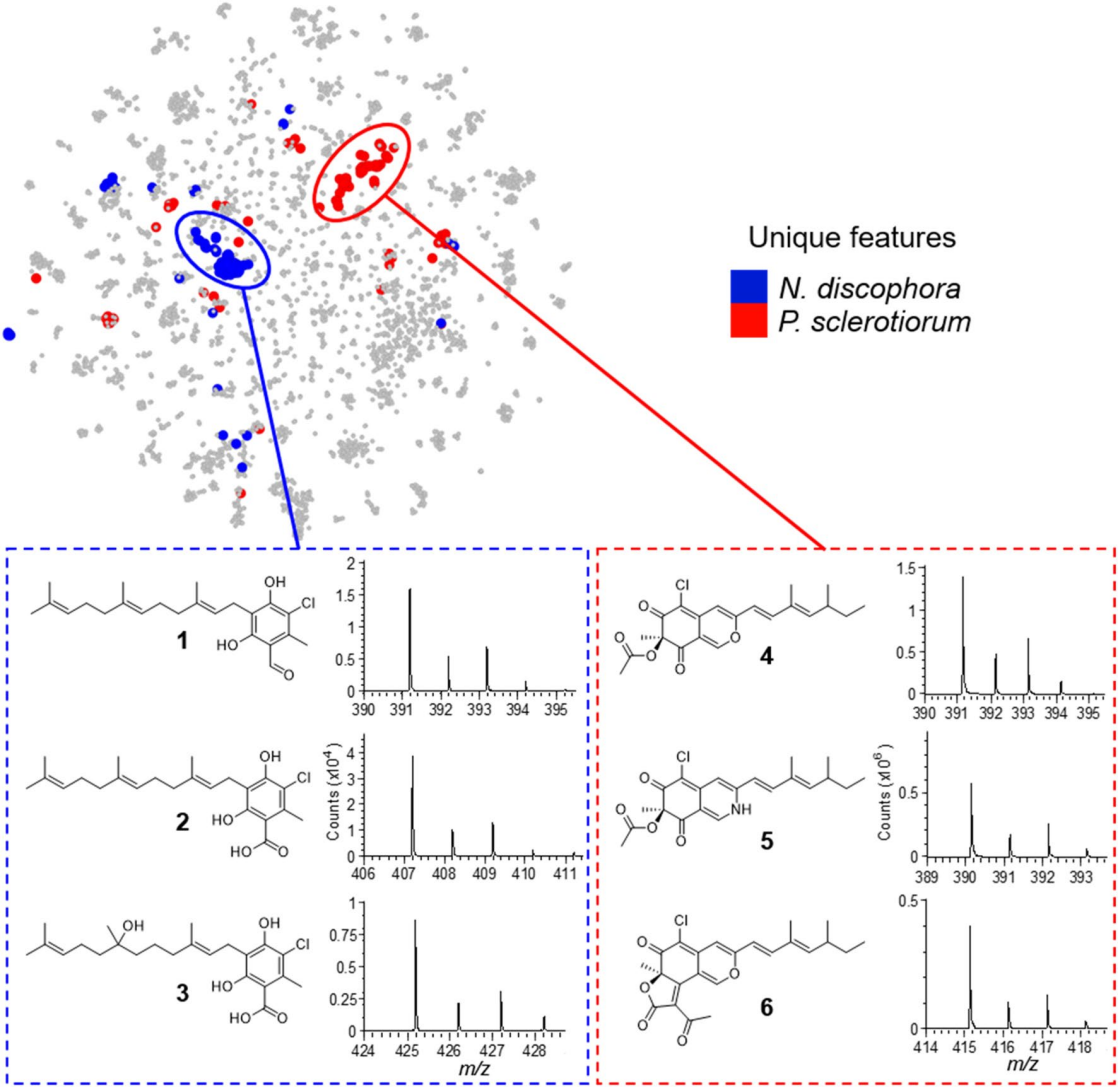
Molecular network obtained for specific specialized metabolomes of *P. sclerotiorum* and *N. discophora* among 130 crude extracts of termite-associated microorganisms (top). MetGem software (https://metgem.github.io/). Chlorinated metabolites have been annotated by comparison of their MS/MS spectra with databases and are depicted with their typical isotopic pattern related to ^37^Cl (bottom).

The ICSN strain collection includes 130 strains of termite mutualistic microorganisms from French Guiana. Each strain was cultivated on solid PDA medium and then extracted by ethyl acetate. The specialized metabolomes of each extract were explored by reverse-phase liquid chromatography coupled with positive electrospray ionization tandem mass spectrometry (RPLC-ESI(+)-MS/MS). The MS/MS data were organized and visualized as a molecular network based on fragmentation spectra homology related to structural homology^27^ with MetGem software^38^ based on t-SNE visualization. We analyzed the production of specialized metabolites with high structural specificity in which nodes were clustered (Fig. 1, top). We identified ilicicolins produced by *Neonectria discophora* SNB-CN63 (blue cluster in Fig. 1) and azaphilones produced by *Penicillium sclerotiorum* SNB-CN111 (red cluster in Fig. 1).

In these two clusters, we observed a specific isotopic pattern at the MS level of chlorinated metabolites via the natural abundance of ^37^Cl isotopes (24.23%) versus ^35^Cl (75.77%)^39^. Thereafter, in the blue cluster, 12 molecules were annotated as ilicicolins by comparison with public or internal databases from previous studies (Table S1, Figure S1)^17,18^. Eight of these molecules bear a chlorine atom, such as LL-Z1272α or ilicicolinal (**1**), ilicicolinic acid A (**2**) and ilicicolinic acid C (**3**) (Fig. 1). In the red cluster, twenty-three azaphilones were annotated using MS/MS spectral comparisons from public or in-house databases (Table S2, Figure S2)^20,40,41^. Among them, 14 are chlorinated, such as sclerotiorin (**4**), sclerotioramine (**5**) or 5-chloroisorotiorin (**6**), which were previously isolated in our group^20,42–44^.

Ilicicolins are reported as intermediates of halogenated metabolites named ascofuranone and ascochlorin, which are chlorinated by a FAD-dependent halogenase (AscD)^45^. In the literature, 28 ilicicolin or acid ilicicolinic scaffolds isolated from natural products have been reported, among which 22 are chlorinated^46^.

In a review article published in 2021, Pavesi et al*.* reported 676 azaphilones, among which 152 contain a chlorine atom^47^. These chlorinated metabolites are included in just four azaphilone subfamilies, i.e., chaetoviridins, falconensins, sclerotiorins and luteusins. Among these scaffolds, only the gene cluster involved in chaetoviridin biosynthesis has been elucidated. In that particular case, the enzyme involved in chlorine addition is also a FAD-dependent halogenase (CazI)^48^.

Hence, we hypothesized that FAD-dependent halogenases catalyze the halogenation of ilicicolins and azaphilones produced by *N. discophora* SNB-CN63 and *P. sclerotiorum* SNB-CN111, respectively^45,48^. To look for these enzymes, we sequenced these two fungal genomes using a combined long- and short-read sequencing approach followed by a hybrid assembly.

### *N. discophora* genome and the ilicicolins biosynthetic pathway

**Figure 2:**
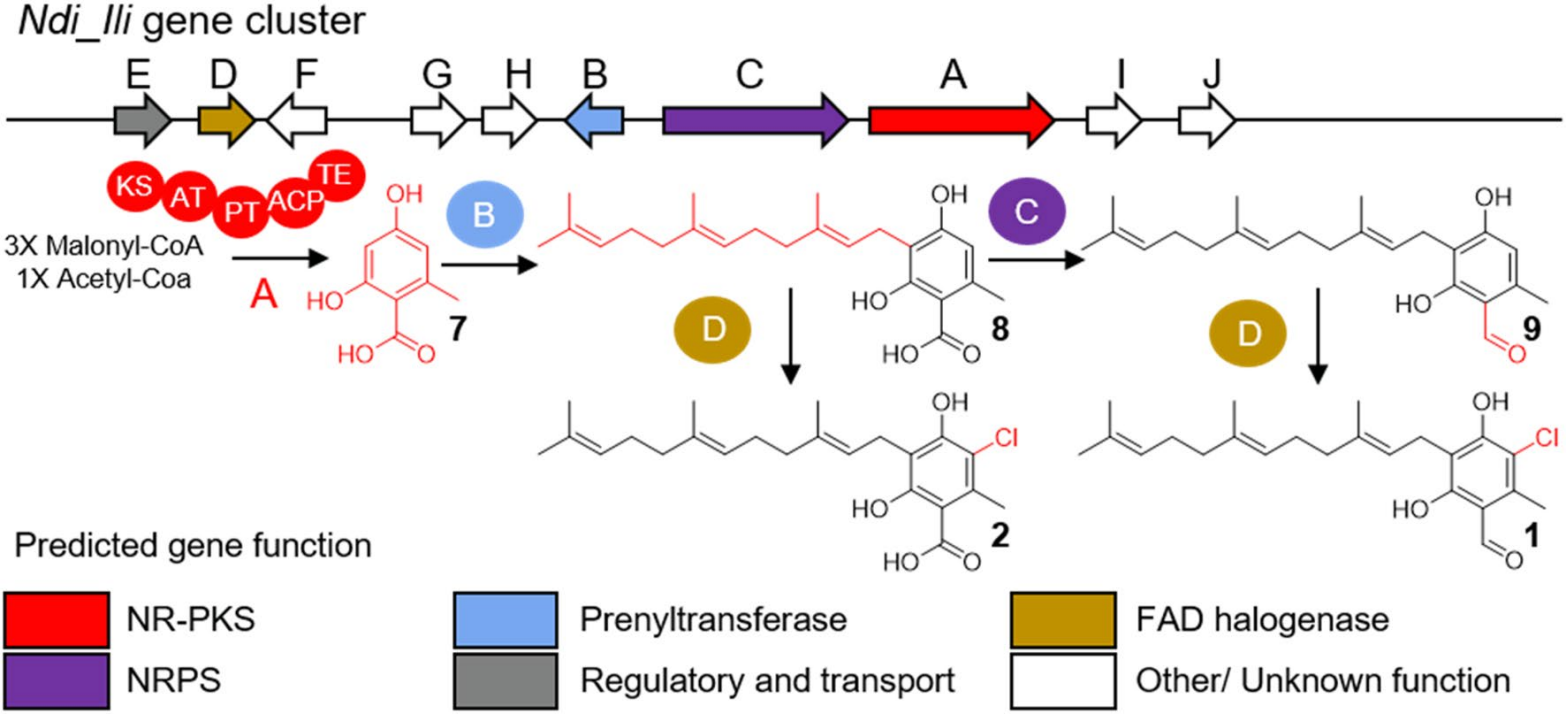
Annotated biosynthetic gene cluster of *N. discophora* SNB-CN63 and related biosynthetic pathways. NR-PKS: nonreducing polyketide synthase, NRPS: nonribosomal peptide synthase. KS: keto-synthase, AT: acyltransferase, PT: product template, ACP: acyl carrier protein, TE: thioesterase. Chemdraw software.

The *N. discophora* genome (ENA accession number: GCA_911649645) was obtained in 26 contigs covering 41.6 Mbp and characterized by a GC content of 54.2%. The completeness of the genome was established at 99.3% using the BUSCO score, and 12,267 genes were predicted (Tables S3, S4)^49^. The contiguity of the genome assembly is characterized by an N50 length of 4.04 Mbp and an L50 of five. We used the antiSMASH pipeline to predict the existence of a biosynthetic gene cluster^31^. Two biosynthetic gene clusters were predicted to include a halogenase as a tailoring enzyme. Sequence comparisons using halogenases from the SwissProt and UniProt databases confirmed that no other putative halogenase was present in the *N. discophora* SNB-CN63 genome. Four genes, a non-reducing polyketide synthase, a prenyltransferase, a non-canonical non-ribosomal peptide synthetase and a halogenase, from candidate cluster *Ndi_Ili* are similar to the ascofuranone biosynthetic gene cluster (59 to 79% similarity, Table S5). Only the halogenase from candidate cluster *Ndi_WSC72* shares 70% similarity with ascD, the ascofuranone halogenase (Table S6). A synteny analysis visualized with *clinker*^50^ further confirm the stronger similarity between *Ndi_Ili* and the ascofuranone biosynthetic gene cluster (Figure S3). Thus, we concluded that *Ndi_Ili* was the best candidate cluster for ilicicolins biosynthesis^45^. This ilicicolin biosynthesis cluster is composed of 10 genes named *Ndi_Ili_A-J* (Fig. 2, Table S5).

The nonreducing polyketide synthase Ndi_Ili_A is composed of five domains, keto-synthase, acyltransferase, product template, acyl carrier protein, and thioesterase, and it is predicted to catalyze orsellinic acid formation (**7**) since it shares 56% identity and 70% similarity with AscC, as described in *Acremonium egyptiacum*^45^. The prenyltransferase Ndis_Ili_B produces grifolic acid (**8**) from orsellinic acid by the addition of a farnesyl group (observed at [M + H]^+^, *m/z* 373.2373, err. 0.0 ppm, C_23_H_32_O_4_), and then a noncanonical nonribosomal peptide synthase Ndi_Ili_C reduces the carboxylic acid function to form ilicicolin B, also named LL-Z 1272β (**9**) ([M + H]^+^, *m/z* 357.2422, err. 0.6 ppm, C_23_H_32_O_3_). Finally, the FAD-dependent halogenase Ndis_Ili_D adds a chlorine atom to the orsellinic scaffold to form Compounds **1** and/or **2** ([M + H]^+^, *m/z* 391.2028, err. 1.7 ppm, C_23_H_31_ClO_3_ and [M + H]^+^, *m/z* 407.1979, err. 1.1 ppm, C_23_H_31_ClO_4_, respectively), indicating some flexibility of halogenase regarding its substrates (Table S7). It is likely that the formation of other compounds previously described in the literature from this strain^45^, such as ilicicolinals and ilicicolinic acids, involves the action of monooxygenase, epoxidase and terpene cyclase acting on the prenyl chain. These enzymes are probably located outside the *Ndis_Ili* gene cluster, as determined by Araki et al. for ascochlorin and ascofuranone^45^.

### The *P. sclerotiorum* genome and azaphilone biosynthetic pathways

**Figure 3:**
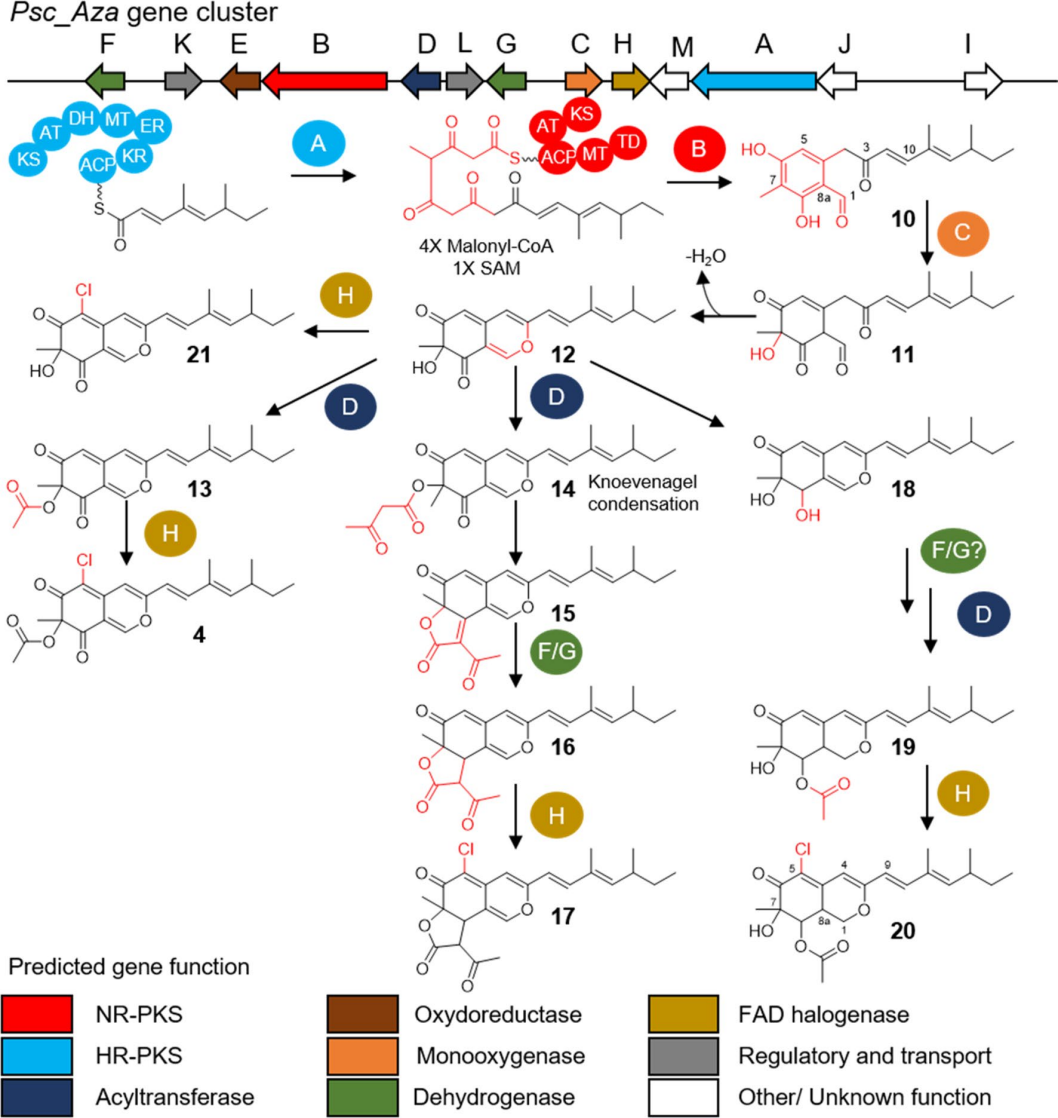
Annotated biosynthetic gene cluster of *P. sclerotiorum* SNB-CN111 and related biosynthetic pathways. NR-PKS: nonreducing polyketide synthase, HR-PKS: highly reducing polyketide synthase. KS: keto-synthase, AT: acyltransferase, DH: dehydratase,MT: methyltransferase, ER: enoylreductase, KR: ketoreductase, ACP: acyl carrier protein, TD: terminal domaine Chemdraw software.

The genome of *P. sclerotiorum* SNB-CN111 (ENA accession number: GCA_911649655) was obtained in 10 contigs covering a total size of 34.7 Mbp and a GC content of 48.3%. The completeness of the genome was established at 98.3% using the BUSCO score, and 12,582 genes were predicted (Tables S3, S4)^49^. The contiguity of the genome assembly is characterized by an N50 length of 4.34 Mbp and an L50 of four. We used the antiSMASH pipeline to annotate a biosynthetic gene cluster. We predicted 15 clusters with polyketide synthase as core enzymes, with three of them containing two polyketide synthases. Only one of these three clusters with two polyketide synthases included a halogenase^31^. This putative azaphilone biosynthesis gene cluster is composed of 13 genes named *Psc_Aza_A-M* (Fig. 3, Tables S8, S9). The Psc_Aza_A protein was identified using a Pfam search as a highly reducing polyketide synthase composed of seven modules: β-ketoacyl synthase, acyltransferase, dehydratase, methyltransferase, enoylreductase, keto-reductase and acyl carrier protein (phosphopantetheine attachment site). This sequence is typical of highly reducing polyketide synthases such as ATEG_07659 (65% identity, 77% similarity) involved in the biosynthesis of azaphilones such as asperfuranone^51^. The *Psc_Aza_B* gene is predicted to encode a nonreducing polyketide synthase composed of five domains: β-ketoacyl synthase, acyltransferase, acyl carrier protein (phosphopantetheine attachment site), methyltransferase and a terminal domain. The enzyme CazM, a nonreducing polyketide synthase involved in chaetoviridin synthesis, is the closest homolog (67% identity, 78% similarity) to Psc_Aza_B^52^. Other azaphilone biosynthetic pathways involving both highly reducing and nonreducing polyketide synthases are described in the literature^47,53^. A synteny cluster comparison using *clinker*^50^ revealed that the polyketide synthase pair involved in other azaphilone biosynthetic pathways (i.e., chaetoviridin, asperfuranone, azanigerone, mitorubrinol and ankaflavin) is conserved and homologous to Psc_Aza_A and Psc_AzaB (Figure S4). These polyketide pairs can operate sequentially, with a highly reducing polyketide synthase producing the first precursor, which is then transferred to nonreducing polyketide synthase and extended (asperfuranone)^54^. They may also operate in a convergent manner with both enzymes being responsible for the biosynthesis of polyketides that are assembled together at later steps (azanigerone)^56^ or in a hybrid manner with both sequential and convergent modes (chaetoviridin)^55^. Finally, Psc_Aza_C, a FAD-dependent monooxygenase, is found in the azaphilone biosynthetic gene cluster^47^. FAD-dependent monooxygenases play a key role in azaphilone synthesis as they are required for the cyclization of the pyran ring. The high sequence similarity of the Psc_Aza_A/Psc_Aza_B polyketide synthases with the CasF/CazM and ATEG_07659/ATEG_07661 couples suggests that the azaphilone biosynthetic pathway is similar to that of asperfuranone or chaetovirin (Figure S4). Moreover, Psc_Aza_A/B/C/D/E/G/H/L are similar to other proteins involved in other azaphilone biosynthetic pathways (Figure S4), strengthening our annotation identification of Psc_Aza as a putative azaphilone biosynthetic pathway responsible for the production of sclerotiorin.

Psc_Aza_A catalyzes the elongation of the 4,6-dimethyl-2,4-octadienal unit, and cyclization is then performed by Psc_Aza_B. The monooxygenase Psc_Aza_C then catalyzes the cyclization of the pyran ring and the formation of the azaphilone scaffold (Fig. 3). This hypothesis about the initiation of the azaphilone biosynthetic pathway is strengthened by the detection in the molecular network of an ion of *m/z* 317.1747 (err. 0.1 ppm) corresponding to the molecular formula C_19_H_24_O_4,_ whose fragmentation spectrum agrees with the structure of metabolite **10** resulting from Psc_Aza_B catalysis (Figure S5). The [M + H]^+^ ion of compound **11** is observed at *m/z* 333.1699 (err. 0.8 ppm), which corresponds to the molecular formula C_19_H_24_O_5_ expected for the biosynthetic intermediate resulting from the biotransformation of metabolite **10** by Psc_Aza_C. The fragmentation spectrum of this ion at *m/z* 333.1699 confirms the proposed structure of intermediate compound **11** (Figure S6). Metabolite **12**, originating from the spontaneous conversion of metabolite **11**, was detected as an [M + H]^+^ ion at *m/z* 315.1594 (err. 1.0 ppm, C_19_H_22_O_4_), leading to a typical fragmentation spectrum from the sclerotiorin scaffold with a fragment at *m/z* 147.0457 (err. − 7.9 ppm) and has not been observed until now for compounds **10** and **11** (Figure S7)^20^. Molecule **12** is also described as an azaphilone intermediate involved in the asperfuranone biosynthesis pathway^56^.

Three biosynthetic pathways are possible with this azaphilone scaffold (**12**). The first pathway leads to the production of sclerotiorin (**4**). It is mediated by the action of an acyl transferase Psc_Aza_D to form compound **13** ([M + H]^+^, *m/z* 357.1705, err. − 2.4 ppm, C_21_H_24_O_5_). Then, the FAD-dependent halogenase Psc_Aza_H leads to the production of molecule **4** ([M + H]^+^, *m/z* 391.1309, err. − 0.6 ppm, C_21_H_23_ClO_5_). The second biosynthetic pathway leads to the formation of isochromophilone I (**17**) and is also initiated by the action of an acyltransferase, probably Psc_Aza_D, which adds an acetoacetate to form compound **14** (not detected) that is spontaneously converted by Knoevenagel condensation into compound **15** ([M + H]^+^, *m/z* 381.1691, err. 1.5 ppm, C_23_H_24_O_5_)^42^*.* The angular lactone is then hydrogenated by the action of Psc_Aza_F or G to form compound **16** ([M + H]^+^, *m/z* 383.1859, err. − 1.6 ppm, C_23_H_26_O_5_), which is then chlorinated by the action of Psc_Aza_H. The third biosynthetic pathway leads to the formation of compound **20**. The formation of molecule **20** requires the action of an oxidoreductase to reduce C-6 ketone and form a hydroxyl, a dehydrogenase (to hydrogenate the C-1/C-8a bond), an acyltransferase and a halogenase. However, without the detection of intermediates between compounds **12** and **19**, it is not possible to define which enzymes are involved and in which order. The enzymes Psc_Aza_D, E, and F/G could be involved in the biosynthesis of metabolites **12** to **19** because of their Pfam domains and their inclusion in the azaphilone biosynthetic gene cluster. Finally, Psc_Aza_H catalyzes the chlorination of molecule **19** ([M + H]^+^, *m/z* 361.2021, err. − 3.2 ppm, C_21_H_28_O_5_) to form compound **20** ([M + H]^+^, *m/z* 395.1626, err. − 1.6 ppm, C_21_H_27_ClO_5_). Notably, compound **21**, the chlorinated analog of intermediate **12** (*m/z* 349.1205, err. − 1.1 ppm, C_19_H_21_ClO_4_) was also detected (Figure S8), suggesting that the FAD-dependent halogenase Psc_Aza_H may catalyze chlorination as soon as the azaphilone scaffold is formed.

Thus, we completed the annotation of the biosynthetic gene cluster of sclerotiorin (**4**) and isochromophilone I (**17**). Both compounds originated from an intermediate with a minimal azaphilone scaffold with a 3,5-dimethyl-1,3-heptadienyl chain (molecule **12**) that is typical of sclerotiorin and its analogs (Fig. 3). The gene coding for a halogenase, Psc_Aza_H, was identified, as well as numerous chlorinated azaphilone intermediates or analogs (Tables S2, S8). The FAD-dependent halogenase may be involved as early as the formation of the azaphilone scaffold since intermediate **21** is the chlorinated analog of compound **12**.

In summary, we identified two clusters of biosynthetic genes that may be responsible for the production of two chlorinated polyketide families: ilicicolins and azaphilones. For each cluster, we annotated a FAD-dependent halogenase. We then applied the OSMAC method to generate structural diversity and to confirm the ability of halogenases in the biosynthetic pathways to introduce various halogens (Cl, Br and I).

### Generation of undescribed halogenated compounds using the OSMAC method

**Figure 4:**
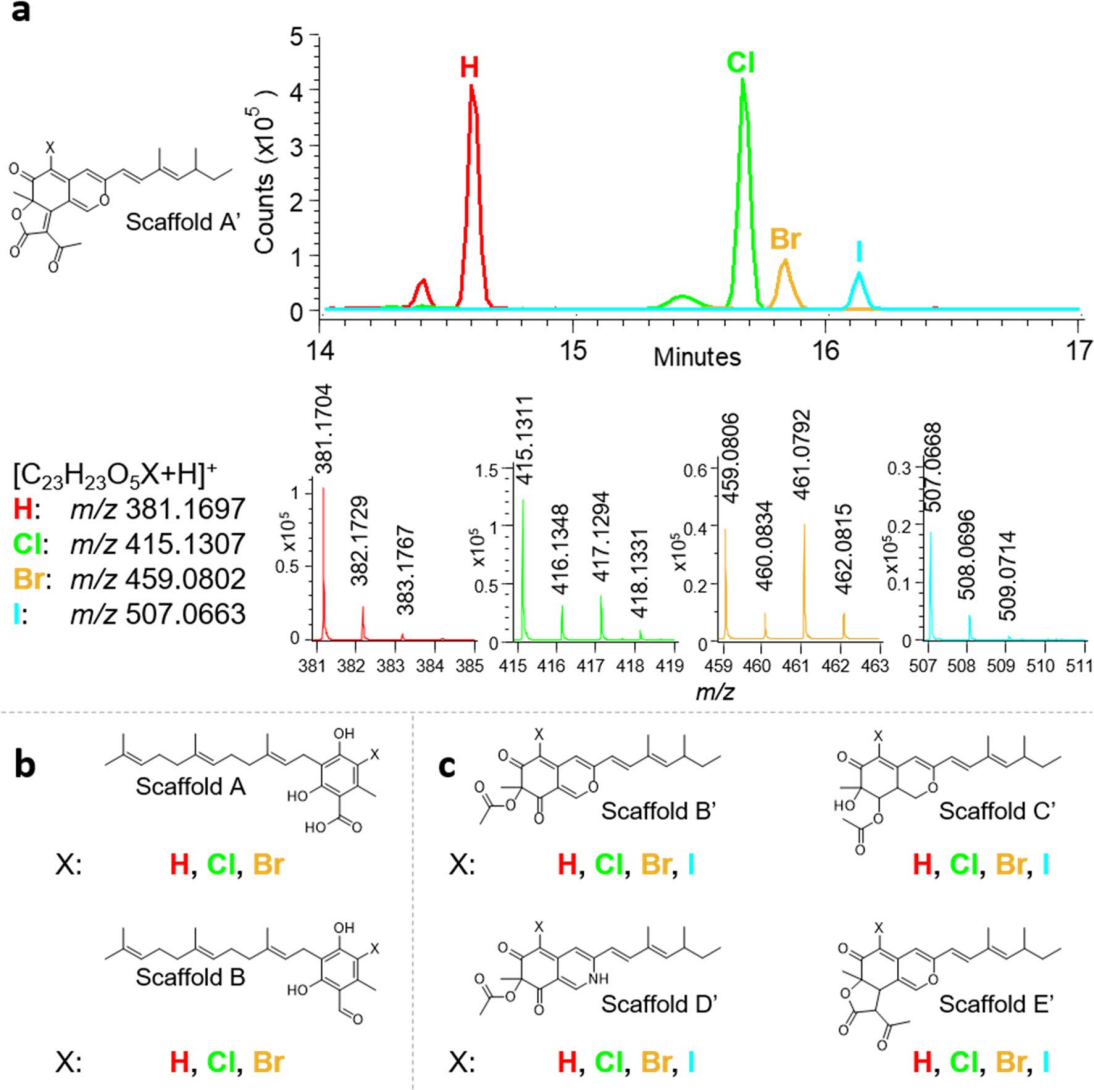
Generation of halogenated azaphilones and ilicicolins by the OSMAC method. (**a**) Extracted ion chromatograms of halogenated azaphilones from scaffold A′ (H, Cl, Br and I) with their isotopic patterns. (**b**) Identified halogenations from Ndi_Ili_D on ilicicolin scaffolds A and B. (**c**) Identified halogenations from Psc_Aza_H on azaphilone scaffolds B′, C′, D′ and E′.

Several studies have highlighted the ability of FAD-dependent halogenases to introduce different halogens, such as Cl, Br and I, into various chemical scaffolds^57–59^. Therefore, we sought to generate new compounds taking into account this biosynthetic possibility from our two sequenced strains: *N. discophora* SNB-CN63 and *P. sclerotiorum* SNB-CN111. For this purpose, we cultivated strains on PDA media supplemented with 10 g L^−1^ NaCl, KBr or KI without affecting microorganism growth. We further analyzed crude extracts by RPLC-ESI(+)-MS/MS to highlight and annotate the major halogenated biosynthesized analogs (Fig. 4a).

We searched the *m/z* values of the protonated species corresponding to the halogenated (Cl, Br and I) metabolites in the MS data. We also examined the isotopic profiles and the retention time (RT), which evolves with the sizes of the halogen substituting H, i.e., H < Cl < Br < I. For example, all azaphilone analogs with H, Cl, Br, and I at the C-5 position for scaffold A′ (Fig. 4a) were detected from *P. sclerotiorum* crude extracts when the corresponding halogen was added to the culture medium. The same approach was applied to *N. discophora* crude extract and demonstrated that the Ndis_Ili_D halogenase is also able to catalyze the addition of Cl and Br to ilicicolins. However, we did not detect any signals related to iodinated species for scaffolds A and B (Fig. 4b, S9, S10). Similar results to scaffold A′ were obtained for azaphilone scaffolds B′, C′, D′, and E′ (Fig. 4c, S11–S14), confirming that Psc_Aza_H can efficiently halogenate azaphilone subfamilies with Cl, Br and I.

**Figure 5:**
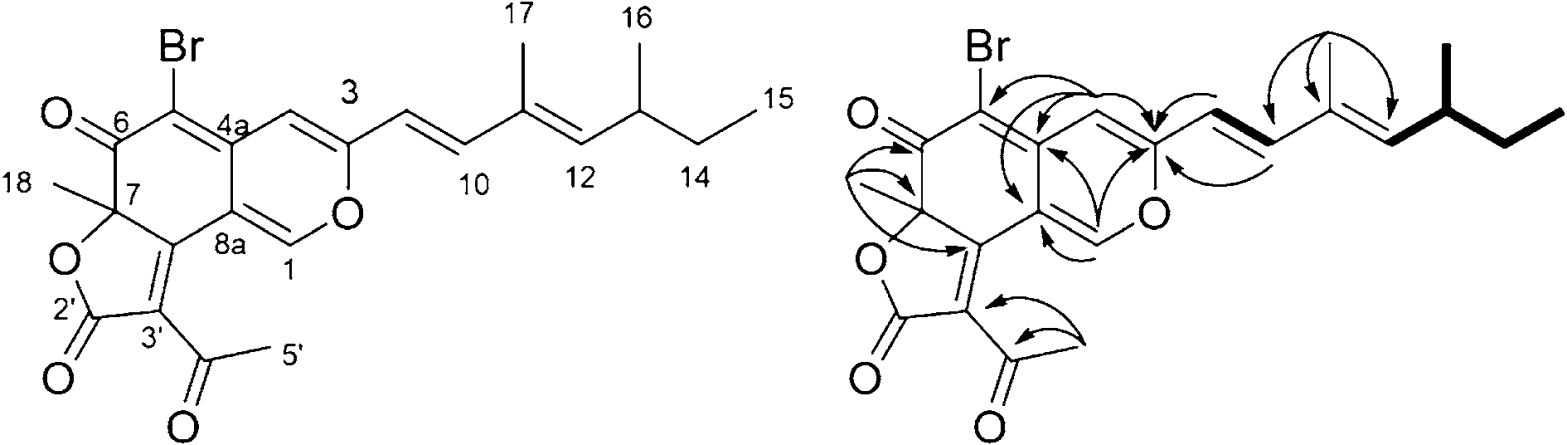
Structural elucidation of compound **22** and 2D RMN correlations observed: ^1^H-^1^H COSY (in bold) and ^1^H-^13^C HMBC (arrows).

We performed a scale-up culture to confirm our structural annotations and to demonstrate that halogenase promiscuity can be exploited to produce sufficient quantities of new and isolable compounds. We used Czapek medium (Czk) to scale up the culture of *P. sclerotiorum* because no organic nitrogen is provided in this medium, thereby leading to a reduced number of produced metabolites. As brominated molecules from scaffolds B′ and E′ have already been described, we focused on scaffold A′^60–62^. Furthermore, nitrogenated azaphilone-like molecules bearing scaffold D′ were not produced in Czk medium, and bromine molecules related to scaffold C′ were not abundant enough. Therefore, compound **22**, which is related to scaffold A′ and has incorporated bromine, was produced and isolated. Compound **22** was obtained as an orange oil and its molecular formula was determined to be C_23_H_23_BrO_5_ based on the ESI-HRMS experiment ([M + H]^+^ peak at *m/z* 459.0798 calcd for C_23_H_23_BrO_5_H^+^, err. 0.7 ppm) (Fig. 5). The azaphilone scaffold was identified by ^13^C NMR of carbon with chemical shifts at δ_C_ of 153.2, 159.7 and 184.5 (C-1, C-3 and C-6, respectively) and validated by HMBC correlations of H1/C-3, C-4a, and C-8a, H4/C-3, C-5, C-8, C-8a and finally H18/C-6, C-7 and C-8. In addition, the HMBC correlations of H-9 and H-10 with C-3 and H-4 with C-3 allowed the side chain to be connected. The lactone moiety was confirmed by the presence of 4 carbons, including 2 carbonyls observed at δ_C_ 195.1 and 169.4, one methene (at δ_C_ 124.8), one methyl group (δ_C_ 30.9) and by HMBC correlations between H-5′ and C-3′ and C-4′. The 3,5-dimethyl-1,3-heptadienyl unit was established by typical correlations of trans-coupled olefinic protons observed in COSY with correlations between H-9/H-10, H-12/H-13/H-16 and H-13/H-14/H-15. As no proton was observed at the C-5 position on the HSQC spectrum, the bromine atom was positioned there (Figures S15-20). This attribution is in accordance with the previously-reported NMR characterization of compounds with the same scaffolds as **15** and **6** (Tables S10, S11)^44,60^. Compound **22** was described for the first time and was named 5-bromoisorotiorin (Figure S21).

To date, the isolation and identification of brominated azaphilones has been described in only three publications, and all of them reported the isolation from marine sponge-derived fungi of the *Penicillium* genus (*P. canescens* and *P. janthinellum*)^62,63^. The authors cultivated these strains with NaBr to obtain these five brominated azaphilones. The new brominated metabolite **22** was also obtained by an OSMAC method using halogenase promiscuity but for the first time from a terrestrial fungus.

In a previous study concerning mutualistic strains isolated from termites, we showed that the PDA extract of SNB-CN111 had antifungal activity against *Trychophyton rubrum*^20^. Therefore, we compared the antimicrobial activity of previously isolated azaphilones 5-chloroisorotiorin (**15**) and sclerotiorin (**4**) with the newly characterized azaphilone 5-bromoisorotiorin (**22**) on the same human pathogen, i.e., *Tricophyton rubrum*. We obtained a minimal inhibitory concentration (MIC) value of 32 μg mL^−1^ for the azaphilone extract and the three individual compounds. Therefore, halogens on azaphilone scaffolds do not seem to modulate the antimicrobial activity of azaphilone, but the promiscuity of Psc_Aza_H halogenase offers the opportunity to generate undescribed natural compounds.

## Conclusion

In this study, which is related to termite-associated microorganisms, we identified two families of halogenated polyketides. Azaphilone and ilicicoline derivatives were identified by metabolomic analysis of a collection of 130 extracts of termite-associated microorganism strains from French Guiana and molecular network reconstruction using t-SNE.

Sequencing and assembly of the whole genomes of the two fungal strains *P. sclerotiorum* SNB-CN111 and *N. discophora* SNB-CN63 were also performed, allowing the annotation of two clusters of biosynthetic genes related to azaphilones and ilicicolines. We showed that the addition of chlorine in both biosynthetic pathways was catalyzed by a halogenase, as described for molecules belonging to the same families^45,47^. Then, we used RPLC-ESI(+)-MS/MS data to annotate biosynthetic intermediates not isolable in the crude extracts or fractions due to low concentrations, reinforcing the biosynthetic pathway hypotheses. These data allowed the identification of halogenated polyketides with high molecular diversity.

FAD-dependent halogenase promiscuity was exploited in our study to generate original chlorinated, brominated or iodinated analogs. One of the brominated compounds was isolated and characterized, and it confirmed our annotations of halogenated compounds in the generated molecular networks.

Finally, the use of halogenases to catalyze environmentally friendly and regioselective halogenations is a promising alternative to classical halogenation processes^58,64^. To this end, it will be relevant to test the ability of the halogenases Psc_Aza_H and Ndi_Ili_D to introduce halogens to chemical backbones different from azaphilone or iliciolin ones, as well as to organic synthesis intermediates such as substituted benzenes. Since halogenase Psc_Aza_H can catalyze halogen addition as soon as the azaphilone scaffold is formed, this enzyme could be integrated into other azaphilone biosynthetic gene clusters to generate more molecular diversity using metabolic engineering.

## Methods

### General experimental procedures

Optical rotations were measured at 20 °C in methanol using an Anton Paar MCP 300 polarimeter in a 100 mm long 350 μL cell (Anton Paar, Graz, Austria) . UV spectra were recorded at 20 °C in MeOH using a PerkinElmer Lambda 5 spectrophotometer (Thermo Fisher scientific, Les Ulis, France). NMR spectra were recorded on Bruker 700 MHz spectrometers (Bruker, Rheinstetten, Germany). The chemical shifts (δ) are reported as ppm based on the solvent signal, and the coupling constants (J) are given in hertz. Preparative HPLC was conducted with a Gilson system equipped with a 322 pumping device, a GX-271 fraction collector, a 171 diode array detector, and a prep ELSII. All solvents were HPLC grade and purchased from Sigma–Aldrich (Saint-Quentin-Fallavier, France).

### Isolation and identification of termite mutualistic microorganisms

The taxonomic marker analyses were performed by BACTUP, France. The identification of the fungi was conducted by amplification of the ITS4 or ITS1 region of ribosomal DNA, and the bacterial isolates were identified on the basis of 16S rDNA sequence analysis. These sequences were compared to the nonredundant nucleotide collection using BLASTN 2.12 (http://www.ncbi.nlm.nih.gov, accessed on August 17, 2021).

The SNB-CN63 strain was isolated from the inside of a *Nasutitermes corniger* termite aerial nest by sampling the inside of an aerial termite mound after its surface sterilization at the location of Sentier des Salines in French Guiana^18,19^. A DNA sample from SNB-CN63 was used for strain identification following nuclear ribosomal internal transcribed spacer region ITS4 sequencing and sequence comparison. The obtained sequence (NCBI accession number KJ023733) was 96% identical over 558 bp to FJ560438.1 from *Neonectria discophora;* therefore, strain SNB-CN63 from the strain library collection at ICSN was identified as *Neonectria discophora*.

The SNB-CN111 strain was isolated as described above from a *Nasutitermes similis* termite aerial nest found in Piste de Saint-Elie in French Guiana^20^. We used the same approach described for SNB-63 to obtain the taxonomic identity of SNB-C111. The obtained ITS sequence (NCBI accession number KJ023726) was 97% identical to KX365203.1 from *Penicillium sclerotium*; therefore, the strain SNB-CN63 from the strain library collection at ICSN was identified as *Penicillium sclerotiorum*.

### Genomic DNA extraction of *Penicillium sclerotiorum* SNB-CN111 and *Neonectria discophora* SNB-CN63

For high molecular weight gDNA extraction, we cultivated the strains *Penicillium sclerotiorum* SNB-CN111 and *Neonectria discophora* SNB-CN63 on PDA medium on a permeable cellophane membrane prepared as described by Fauchery et al*.*^65^. The cellophane membranes were trimmed to the size of the petri dish, placed into boiling distilled water containing EDTA (1 g L^−1^) for 20 min to permeabilize the membrane, rinsed four times in a large container with distilled water and autoclaved. After cultivation for seven days at 26 °C, microorganisms were removed from the cellophane membrane, snap frozen in liquid nitrogen and milled using a mortar and pestle.

gDNA was extracted from microorganisms using NucleoBond Buffer Set III and AXG 100 (Macherey–Nagel, Hoerdt, France) with slight modifications: 250 mg of milled mycelium was resuspended in 5 mL of buffer G3 at 37 °C for 14 h, 1.2 mL of buffer G4 was added, and the solution was gently mixed and then incubated at 50 °C for 4 h. The sample was clarified through centrifugation at 5000*g* for 5 min. After ethanol precipitation, the samples were re-dissolved in 100 µL of nuclease-free water. Purity was measured using a Nanodrop 2000 spectrophotometer, and DNA quantity was measured using a Qubit dsDNA BR assay kit (according to the manufacturer’s recommendation) and a Qubit fluorometer. The integrity of DNA was assessed by electrophoresis on a 0.7% agarose gel in TBE 0.5X. We performed an additional size selection and cleanup for *N. discophora* SNB-CN63 DNA using the Circulomics Short Read Eliminator kit (www.circulomics.com).

### Whole genome sequencing and hybrid assembly

*P. sclerotiorum* SNB-CN111 and *N. discophora* SNB-CN63 DNA samples were prepared for shotgun sequencing according to native barcoding expansion (EXP-NBD-103) and the 1D Native barcoding genomic DNA protocol (SQK-LSK109). We loaded 50 femtomoles of purified pooled library on a FLO-MIN111 flowcell (R10.3, Oxford Nanopore Technologies) and ran the sequencing for 72 h.

Short-read DNA sequencing was outsourced to Novogene (https://en.novogene.com/). The same DNA used for long-read sequencing was fragmented (mean size of 350 bp) and sequenced using a paired-end strategy on an Illumina NovaSeq 6000 to obtain two 150 nt reads per DNA fragment. Overall, we obtained an estimated coverage of 304X for CN63 (143X and 161X for short and long reads, respectively) and 191X for CN111 (38X and 153X for short and long reads, respectively).

We performed base calling and adapter trimming using Guppy 3.2.10 for long reads and checked raw read quality using NanoPlot^66^. Finally, we used WenganM for genome assembly^67^. The sequence datasets generated and/or analyzed during the current study are available in the EMBL Nucleotide Sequence Database (ENA) and National Center for Biotechnology Information (NCBI), SNB-CN63 (GCA_911649645) and SNB-CN111 (GCA_911649655) (https://www.ebi.ac.uk/ena/data/view/PRJEB46500). Individual gene sequences for the biosynthetic clusters are available in the EMBL Nucleotide Sequence Database (ENA) at http://www.ebi.ac.uk/ena/data/view/OU452329-OU452351.

### Culture and extraction of microorganisms

All strains were cultivated on solid PDA medium at 26 °C for 15 days on four Petri dishes of 10 cm diameter (85 cm^2^). For the OSMAC experiments, the microorganisms were cultivated under identical conditions on four Petri dishes of 10 cm diameter (85 m^2^) with PDA medium supplemented with 10 g L^−1^ NaCl, KBr and KI. For the large-scale culture of *P. sclerotiorum,* the microorganism was cultivated under identical conditions on 60 Petri dishes of 10 cm diameter (85 m^2^) with Czapek medium supplemented with 10 g L^−1^ of KBr. The contents of the Petri dishes were transferred into a large container and macerated with EtOAc for 24 h. Insoluble residues were removed via filtration, and the organic phase was washed three times with an equivalent volume of water (H_2_O), dried with anhydrous solid Na_2_SO_4_ and then evaporated using a rotary evaporator under reduced pressure and a temperature of 30 °C.

### Halogenated compound isolation and characterization

The whole crude extract of *P. sclerotiorum* cultivated on Czapek supplemented with 10 g KBr L^−1^ (1.2 g) was fractionated by reversed-phase flash chromatography (Grace Reveleris, Grace, Maryland, USA) using a 120 g C18 column and ultraviolet (UV) and evaporative light scattering detectors (ELSDs). A linear gradient was formed from a mixture of solvent A (H_2_O/formic acid 99.9/0.1) and solvent B (acetonitrile/formic acid (99.9/0.1)), from 5% of solvent B to 100% in 40 min, with a flow rate of 80 mL min^−1^, followed by a second gradient of solvent B and solvent C (methylene chloride), from 50 to 100% of C in 15 min, with a flow rate of 80 mL min^−1^, to generate eight fractions labeled F1 to F8. Fraction of interest F6 (60 mg) was submitted to preparative HPLC. Fraction F6 was purified using an isocratic condition of 80% solvent B for 40 min and led to the isolation of the new compound **22** (2.5 mg, *t*_*R*_ = 28.6 min).

5-Bromoisorotiorin (**22**): orange amorphous oil; [α]^20^_D_ 330 (*c* 0.1 g L^−1^, MeOH), UV (MeOH) λmax (ε), 380.

(5 800 L mol^−1^ cm^−1^), 422 (6 000 L mol^−1^ cm^−1^), ^1^H NMR (700 MHz, DMF) δH 8.82 (1H, s, H-1), 6.98 (1H, s, H-4), 6.78 (1H, d, J = 16.1 Hz, H-9), 7.26 (1H, d, J = 16.2 Hz, H-10), 5.87 (1H, d, J = 10.2 Hz, H-12), 2.55 (1H, m, H-13), 1.45 (1H, m, H-14a), 1.34 (1H, m, H-14b), 0.87 (3H, t, J = 7.4 Hz, H-15), 1.02 (3H, d, J = 6.7 Hz, H-16), 1.92 (3H, s, H-17), 1.72 (3H, s, H-18), 2.57 (3H, s, H-5′), ^13^C NMR (700 MHz, DMF) δ_C_ 153.2 (CH, C-1), 159.7 (C, C-3), 109.5 (CH, C-4), 143.4 (C, C-4a), 100.8 (C, C-5), 184.5 (C, C-6), 88.4 (C, C-7), 164.2 (C, C-8), 112.1 (C, C-8a), 118.1 (CH, C-9), 143.6 (CH, C-10), 133.9 (C, C-11), 149.1 (CH, C-12), 35.9 (CH, C-13), 30.3 (CH_2_, C-14), 12.5 (CH_3_, C-15), 20.8 (CH_3_, C-16), 12.9 (CH_3_, C-17), 26.4 (CH_3_, C-18), 169.4 (CH_2_, C-2′), 124.8 (C, C-3′), 195.2 (C, C-4′), 30.5 (CH_3_, C-5′), ESI-HRMS *m/z* [M + H]^+^ 459.0798 (calcd for C_23_H_22_BrO_5_H^+^, 459.0802, err. -0.7 ppm).

### RPLC-ESI(+)-MS/MS analysis

Crude extracts of all SNB-CN strains cultivated and extracted as previously described together with fractions from *Penicillium sclerotiorum* SNB-CN111 were prepared at 1 mg mL^−1^ in methanol and filtered through a 0.45 µm PTFE membrane. RPLC-ESI(+)-MS/MS experiments were performed with a 1260 Prime HPLC (Agilent Technologies, Waldbronn, Germany) coupled with an Agilent 6540 Q-ToF (Agilent Technologies, Waldbronn, Germany) tandem mass spectrometer. RPLC separation was achieved with an Accucore RP-MS column (100 × 2.1 mm, 2.6 μm, Thermo Scientific, Les Ulis, France) with a mobile phase consisting of H_2_O/formic acid (99.9/0.1) (A)—acetonitrile/formic acid (99.9/0.1) (B). The column oven was set at 45 °C. Compounds were eluted at a flow rate of 0.4 mL min^−1^ with a gradient from 5% B to 100% B in 20 min and then maintained at 100% B for 3 min. The injection volume was fixed at 5 μL for all analyses. For the electrospray ionization source, mass spectra were recorded in positive ion mode with the following parameters: gas temperature 325 °C, drying gas flow rate 10 L min^−1^, nebulizer pressure 30 psi, sheath gas temperature 350 °C, sheath gas flow rate 10 L min^−1^, capillary voltage 3500 V, nozzle voltage 500 V, fragmentor voltage 130 V, skimmer voltage 45 V, and octopole 1 RF voltage 750 V. Internal calibration was achieved with two calibrants, purine and hexakis (1 h,1 h,3 h-tetrafluoropropoxy)phosphazene (*m/z* 121.0509 and *m/z* 922.0098), providing a high mass accuracy better than 10 ppm. The data-dependent MS/MS events were acquired for the five most intense ions detected by full-scan MS, from the 200–1000 m*/z* range, and above an absolute threshold of 1000 counts. Selected precursor ions were fragmented at a fixed collision energy of 30 eV and with an isolation window of 1.3 amu. The mass range of the precursor and fragment ions was set as *m/z* 200–1000.

Isolated compounds from *Penicillium sclerotiorum* SNB-CN111 fractions were prepared at 0.05 mg mL^−1^ in methanol and filtered through a 0.45 µm PTFE membrane. The isolated compounds were analyzed according to the same procedure.

### Data processing and analysis

The data files were converted from the .d standard data format (Agilent Technologies) to.mzXML format using MSConvert software, part of the ProteoWizard package 3.0^68^. All .mzxml files were processed using MZmine2v53 as previously described^69,70^. Mass detection was realized with an MS1 noise level of 1000 and an MS/MS noise level of 0. The ADAP chromatogram builder was employed with a minimum group size of scans of 3, a group intensity threshold of 1000, a minimum highest intensity of 1000, and an *m/z* tolerance of 0.008 (or 20 ppm). Deconvolution was performed with the ADAP wavelet algorithm according to the following settings: S/N threshold = 10, minimum features height = 1000, coefficient/area threshold = 10, peak duration range 0.01–1.5 min, and *t*_*R*_ wavelet range 0.00–0.04 min. MS/MS scans were paired using an *m/z* tolerance range of 0.05 Da and *t*_*R*_ tolerance range of 0.5 min. Isotopologues were grouped using the isotopic peak grouper algorithm with an *m/z* tolerance of 0.008 (or 20 ppm) and a *t*_*R*_ tolerance of 0.2 min. Peaks were filtered using a feature list row filter, keeping only peaks with MS/MS scans (GNPS). Peak alignment was performed using the join aligner with an *m/z* tolerance of 0.008 (or 20 ppm), a weight for *m/z* at 20, a retention time tolerance of 0.2 min, and weight for *t*_*R*_ at 50. The MGF file and the metadata were generated using the export/submit to GNPS option.

Molecular networks were calculated and visualized using MetGem 1.34 software^38^, and MS/MS spectra were window-filtered by choosing only the top 6 peaks in the ± 50 Da window throughout the spectrum. The data were filtered by removing all peaks in the ± 17 Da range around the precursor *m/z*. The *m/z* tolerance windows used to find the matching peaks were set to 0.02 Da, and cosine scores were kept in consideration for spectra sharing at least 2 matching peaks. The number of iterations, perplexity, learning rate, and early exaggeration parameters were set to 5000, 25, 200, and 12, respectively, for the t-SNE view.

Figures were generated using R and related packages (ggplot2, Rcolorbrewer, and gridextra), MetGem export function, and ChemDraw Professional 16.0 (PerkinElmer). NMR spectra were processed and analyzed using TopSpin 3.6.2 (Bruker, Rheinstetten, Germany).

### Biological assays

Pure isolated compounds were tested on the human pathogenic microorganism *Trichophyton rubrum* (SNB-TR1). This clinical isolate was provided by Phillipe Loiseau (University Paris-Saclay, Châtenay-Malabry, France). Extracts, fractions, and pure compounds were tested according to the reference protocol of the European Committee on Antimicrobial Susceptibility Testing^71^. The minimal inhibitory concentration value was obtained after 72 h. The MIC values reported refer to the lowest concentration preventing visible growth in the wells. Pure compounds were tested at concentrations ranging from 256 to 0.5 μg mL^−1^. All assays were conducted in duplicate. Itraconazole (MIC = 4 µg mL^−1^) was used as a positive control.

## Supplementary Information

Supplementary Information.

## Data Availability

The datasets generated and/or analyzed during the current study are available in the [EMBL Nucleotide Sequence Database (ENA)] repository, [https://www.ebi.ac.uk/ena/data/view/PRJEB46500, accession number PRJEB46500] and [http://www.ebi.ac.uk/ena/data/view/OU452329-OU452351]; and in the [National Center for Biotechnology Information (NCBI)] repository [http://www.ncbi.nlm.nih.gov, accession numbers KJ023733, KJ023726, GCA_911649645 and GCA_911649655].

## References

[1] Chiocchio I (2018). Screening of a hundred plant extracts as tyrosinase and elastase inhibitors, two enzymatic targets of cosmetic interest. Ind. Crops Prod..

[2] Davison EK, Brimble MA (2019). Natural product derived privileged scaffolds in drug discovery. Curr. Opin. Chem. Biol..

[3] Aleku GA (2017). A reductive aminase from *Aspergillus oryzae*. Nat. Chem..

[4] Stork NE, McBroom J, Gely C, Hamilton AJ (2015). New approaches narrow global species estimates for beetles, insects, and terrestrial arthropods. Proc. Natl. Acad. Sci. U.S.A..

[5] Brune A (2014). Symbiotic digestion of lignocellulose in termite guts. Nat. Rev. Microbiol..

[6] Beemelmanns C, Guo H, Rischer M, Poulsen M (2016). Natural products from microbes associated with insects. J. Org. Chem..

[7] Dillon RJ, Dillon VM (2004). The gut bacteria of insects: nonpathogenic interactions. Annu. Rev. Entomol..

[8] Matsui T, Tanaka J, Namihira T, Shinzato N (2012). Antibiotics production by an actinomycete isolated from the termite gut. J. Basic Microbiol..

[9] Zhang Y (2013). Antifungal activities of metabolites produced by a termite-associated *Streptomyces canus* BYB02. J. Agric. Food Chem..

[10] Mevers E, Chouvenc T, Su NY, Clardy J (2017). Chemical interaction among termite-associated microbes. J. Chem. Ecol..

[11] Kim KH, Ramadhaer TR, Beemelmanns C, Cao S, Poulsen M, Currie CR, Clardy J (2014). Natalamycin A, an ansamycin from a termite-associated *Streptomyces* sp. Chem. Sci..

[12] Carr G (2012). Microtermolides A and B from termite-associated *Streptomyces* sp. and structural revision of Vinylamycin. Org. Lett..

[13] Jao W-H (2018). Trichodermides A–E: New peptaibols isolated from the Australian Termite Nest-Derived Fungus *Trichoderma virens* CMB-TN16. J. Nat. Prod..

[14] Nirma C, Eparvier V, Stien D (2013). Antifungal agents from Pseudallescheria boydii SNB-CN73 isolated from a *Nasutitermes* sp. termite. J. Nat. Prod..

[15] Nirma C, Eparvier V, Stien D (2015). Reactivation of antibiosis in the entomogenous fungus *Chrysoporthe* sp. SNB-CN74. J. Antibiot..

[16] Sorres J, Nirma C, Eparvier V, Stien D (2017). Pseudallicins A-D, four complex ovalicin derivatives from *Pseudallescheria boydii* SNB-CN85. Org. Lett..

[17] Sorres J, Nirma C, Eparvier V, Stien D (2017). Tyroscherin and tyroscherin analogs from Pseudallescheria boydii SNB-CN85 isolated from termite *Termes* cf. *hispaniolae*. Phytochem. Lett..

[18] Nirma C, Eparvier V, Stien D (2015). Antibacterial ilicicolinic acids C and D and ilicicolinal from *Neonectria discophora* SNB-CN63 isolated from a termite nest. J. Nat. Prod..

[19] Sorres J, Sabri A, Brel O, Stien D, Eparvier V (2018). Ilicicolinic acids and ilicicolinal derivatives from the fungus *Neonectria discophora* SNB-CN63 isolated from the nest of the termite *Nasutitermes corniger* found in French Guiana show antimicrobial activity. Phytochemistry.

[20] Hebra T (2021). Dereplication, annotation, and characterization of 74 potential antimicrobial metabolites from *Penicillium Sclerotiorum* using t-SNE Molecular Networks. Metabolites.

[21] Gribble GW (2015). A recent survey of naturally occurring organohalogen compounds. Environ. Chem..

[22] Wang C, Lu H, Lan J, Zaman KHA, Coa S (2021). A review: Halogenated compounds from marine fungi. Molecules.

[23] Mapari SAS, Thrane U, Meyer AS (2010). Fungal polyketide azaphilone pigments as future natural food colorants?. Trends Biotechnol..

[24] Chen C (2020). Recent advances in chemistry and biology of azaphilones. RSC Adv..

[25] Nihei C, Fukai Y, Kita K (2002). Trypanosome alternative oxidase as a target of chemotherapy. Biochim. Biophys. Acta BBA Mol. Basis Dis..

[26] Wolfender J-L, Litaudon M, Touboul D, Queiroz EF (2019). Innovative omics-based approaches for prioritisation and targeted isolation of natural products—New strategies for drug discovery. Nat. Prod. Rep..

[27] Wang M (2016). Sharing and community curation of mass spectrometry data with Global Natural Products Social Molecular Networking. Nat. Biotechnol..

[28] Noda-Garcia L, Tawfik DS (2020). Enzyme evolution in natural products biosynthesis: Target- or diversity-oriented?. Curr. Opin. Chem. Biol..

[29] Lu Y, Wang Y, Zhu W (2010). Nonbonding interactions of organic halogens in biological systems: Implications for drug discovery and biomolecular design. Phys. Chem. Chem. Phys..

[30] van Dijk EL, Jaszczyszyn Y, Naquin D, Thermes C (2018). The third revolution in sequencing technology. Trends Genet..

[31] Blin K (2021). antiSMASH 6.0: Improving cluster detection and comparison capabilities. Nucleic Acids Res..

[32] Lin F-Y, MacKerell AD (2017). Do halogen–hydrogen bond donor interactions dominate the favorable contribution of halogens to ligand–protein binding?. J. Phys. Chem. B.

[33] Mendez L, Henriquez G, Sirimulla S, Narayan M (2017). Looking back, looking forward at halogen bonding in drug discovery. Molecules.

[34] Bode HB, Bethe B, Höfs R, Zeeck A (2002). Big effects from small changes: possible ways to explore nature’s chemical diversity. Chembiochem. Eur. J. Chem. Biol..

[35] Schiewe H-J, Zeeck A (1999). Cineromycins, γ-butyrolactones and ansamycins by analysis of the secondary metabolite pattern created by a single strain of *Strepomyces*. J. Antibiot..

[36] Brel O (2020). Paecilosetin derivatives as potent antimicrobial agents from *Isaria farinosa*. J. Nat. Prod..

[37] Hoang TPT (2018). Metabolomics-driven discovery of meroterpenoids from a mussel-derived *Penicillium ubiquetum*. J. Nat. Prod..

[38] Olivon F (2018). MetGem software for the generation of molecular networks based on the t-SNE algorithm. Anal. Chem..

[39] de Laeter JR (2003). Atomic weights of the elements. Review 2000 (IUPAC Technical Report). Pure Appl. Chem..

[40] Tsugawa H (2015). MS-DIAL: Data-independent MS/MS deconvolution for comprehensive metabolome analysis. Nat. Methods.

[41] Horai H (2010). MassBank: A public repository for sharing mass spectral data for life sciences. J. Mass Spectrom..

[42] Fielding HC, Robertson A, Travers RB, Whalley WB (1957). The chemistry of fungi. Part XXXII. The oxidation of sclerotioramine and the structure of sclerotiorin. J. Chem. Soc. Resumed.

[43] Eade RA, Page H, Robertson A, Turner K, Whalley WB (1957). The chemistry of fungi. Part XXVIII. Sclerotiorin and its hydrogenation products. J. Chem. Soc. Resumed.

[44] Pairet L, Wrigley SK, Reynolds IE, Hayes MA (1995). Azaphilones with endothelin receptor binding activity produced by *Penicillium sclerotiorum*: Taxonomy, fermentation, isolation, structure elucidation and biological activity. J. Antibiot..

[45] Araki Y (2019). Complete biosynthetic pathways of ascofuranone and ascochlorin in *Acremonium egyptiacum*. Proc. Natl. Acad. Sci. USA.

[46] Database for chemical compounds, bibliographic data and chemical reactions. https://www.reaxys.com (accessed on November 22, 2021).

[47] Pavesi C (2021). Biosynthesis of azaphilones: A review. Nat. Prod. Rep..

[48] Sato M (2016). Combinatorial generation of chemical diversity by redox enzymes in chaetoviridin biosynthesis. Org. Lett..

[49] Simão FA, Waterhouse RM, Ioannidis P, Kriventseva EV, Zdobnov EM (2015). BUSCO: Assessing genome assembly and annotation completeness with single-copy orthologs. Bioinformatics.

[50] Gilchrist CLM, Chooi Y-H (2021). clinker & clustermap.js: automatic generation of gene cluster comparison figures. Bioinformatics.

[51] Huang X, Zhang W, Tang S, Wei S, Lu X (2020). Collaborative biosynthesis of a class of bioactive azaphilones by two separate gene clusters containing four PKS/NRPSs with transcriptional crosstalk in fungi. Angew. Chem. Int. Ed..

[52] Winter JM (2015). Biochemical and structural basis for controlling chemical modularity in fungal polyketide biosynthesis. J. Am. Chem. Soc..

[53] Williams K, Greco C, Bailey AM, Willis CL (2021). Core steps to the azaphilone family of fungal natural products. ChemBioChem.

[54] Zabala AO, Xu W, Chooi Y-H, Tang Y (2012). Characterization of a silent azaphilone gene cluster from *Aspergillus niger* ATCC 1015 reveals a hydroxylation-mediated pyran-ring formation. Chem. Biol..

[55] Winter JM (2012). Identification and characterization of the chaetoviridin and chaetomugilin gene cluster in *Chaetomium globosum* reveal dual functions of an iterative highly-reducing polyketide synthase. J. Am. Chem. Soc..

[56] Chiang Y-M (2009). A Gene cluster containing two fungal polyketide synthases encodes the biosynthetic pathway for a polyketide, asperfuranone, in *Aspergillus nidulans*. J. Am. Chem. Soc..

[57] Mori S, Pang AH, Thamban Chandrika N, Garneau-Tsodikova S, Tsodikov OV (2019). Unusual substrate and halide versatility of phenolic halogenase PltM. Nat. Commun..

[58] Fraley AE (2017). Function and structure of MalA/MalA′, Iterative halogenases for late-stage C-H functionalization of indole alkaloids. J. Am. Chem. Soc..

[59] Matsuzaki K (1998). New brominated and halogen-less derivatives and structure-activity relationship of azaphilones inhibiting gp120-CD4 binding. J. Antibiot. (Tokyo).

[60] Paranjape SR (2015). Azaphilones inhibit tau aggregation and dissolve tau aggregates in vitro. ACS Chem. Neurosci..

[61] Somoza AD, Lee K-H, Chiang Y-M, Oakley BR, Wang CCC (2012). Reengineering an azaphilone biosynthesis pathway in *Aspergillus nidulans* to create lipoxygenase inhibitors. Org. Lett..

[62] Frank M (2019). Brominated azaphilones from the sponge-associated fungus *Penicillium canescens* Strain 4.14.6a. J. Nat. Prod..

[63] Chen M (2019). NaBr-induced production of brominated azaphilones and related tricyclic polyketides by the marine-derived fungus *Penicillium janthinellum* HK1-6. J. Nat. Prod..

[64] Latham J, Brandenburger E, Shepherd SA, Menon BRK, Micklefield J (2018). Development of halogenase enzymes for use in synthesis. Chem. Rev..

[65] Fauchery, L., Uroz, S., Buée, M. & Kohler, A. Purification of fungal high molecular weight genomic DNA from environmental samples. in *Fungal Genomics,* (eds. de Vries, R. P., Tsang, A. & Grigoriev, I. V.) vol. 1775 21–35 (Springer New York, 2018). 10.1007/978-1-4939-7804-5_3 29876806

[66] De Coster W, D’Hert S, Schultz DT, Cruts M, Van Broeckhoven C (2018). NanoPack: visualizing and processing long-read sequencing data. Bioinformatics.

[67] Di Genova A, Buena-Atienza E, Ossowski S, Sagot M-F (2021). Efficient hybrid de novo assembly of human genomes with WENGAN. Nat. Biotechnol..

[68] Chambers MC (2012). A cross-platform toolkit for mass spectrometry and proteomics. Nat. Biotechnol..

[69] Pluskal T, Castillo S, Villar-Briones A, Orešič M (2010). MZmine 2: Modular framework for processing, visualizing, and analyzing mass spectrometry-based molecular profile data. BMC Bioinformatics.

[70] Olivon F, Grelier G, Roussi F, Litaudon M, Touboul D (2017). MZmine 2 Data-Preprocessing to enhance molecular networking reliability. Anal. Chem..

[71] European Committee on Antimicrobial Susceptibility Testing: EUCAST. Available online: https://eucast.org/ (accessed on April 11, 2020).

