# Supplementary material for: Combining OSMAC, metabolomic and genomic methods for the production and annotation of halogenated azaphilones and ilicicolins in termite symbiotic fungi
Source: Sci Rep. 2022 Oct 15;12:17310. doi: 10.1038/s41598-022-22256-3 (PMC9569342; doi:10.1038/s41598-022-22256-3)
Supplement: Supplementary file 1 — Supplementary Information. [file 41598_2022_22256_MOESM1_ESM.pdf]

Combining OSMAC, metabolomic and genomic methods for the production and annotation of halogenated azaphilones and ilicicolins in termite symbiotic fungi

Téo Hebra[1], Nicolas Pollet[2], David Touboul[1,3]*, Véronique Eparvier[1]*

[1] Université Paris-Saclay, CNRS, Institut de Chimie des Substances Naturelles, UPR 2301, 91198, Gif-sur-Yvette, France.
[2] Université Paris-Saclay, CNRS, IRD, Évolution Génomes Comportement & Écologie, 91198 Gif-sur-Yvette, France.
[3] Laboratoire de Chimie Moléculaire (LCM), CNRS UMR 9168, École Polytechnique, Institut Polytechnique de Paris, 91128 Palaiseau, France

*emails: veronique.eparvier@cnrs.fr; david.touboul@cnrs.fr

Supplementary data

Figure S1: Dereplicated ilicicolins structures with their theoretical exact masses and experimental fragmentation spectrum comparison with databases. * represents the amount of H_2O losses during ionization. 4
Figure S2: Dereplicated azaphilone structures with their theoretical exact masses and experimental fragmentation spectrum comparison with databases. 8
Figure S3: Cluster comparison of azaphilone biosynthetic gene clusters described in literature with the putative azaphilone biosynthetic gene cluster of Penicillium sclerotiorum SNB-CN111. Only functions of tailoring enzyme related to P. sclerotiorum are annotated. BGC identifier are accession number from MIBIG repository. 16
Figure S4: Cluster comparison of azaphilone biosynthetic gene clusters described in literature with the putative azaphilone biosynthetic gene cluster of Penicillium sclerotiorum SNB-CN111. Only functions of tailoring enzyme related to P. sclerotiorum are annotated. BGC identifier are accession number from MIBIG repository. 19
Figure S5: MS/MS spectra of [**10**+H]$^+$ and the corresponding fragmentation scheme. 20
Figure S6: MS/MS spectra of [**11**+H]$^+$ and the corresponding fragmentation scheme. 20
Figure S7: MS/MS spectra of [**12**+H]$^+$ and the corresponding fragmentation scheme. 21
Figure S8: Comparison of intermediates **12** and **21** fragmentation spectra with their common neutral losses corresponding to H/Cl differences. 21
Figure S9: Extracted ion chromatogram of halogenated ilicicolins from scaffold A (H, Cl and Br) with their isotopic patterns. 23
Figure S10: Extracted ion chromatogram of halogenated ilicicolins from scaffold B (H, Cl and Br) with their isotopic patterns. 23
Figure S11: Extracted ion chromatogram of halogenated azaphilones from scaffold B' (H, Cl, Br and I) with their isotopic patterns. 24
Figure S12: Extracted ion chromatogram of halogenated azaphilones from scaffold C' (H, Cl, Br and I) with their isotopic patterns. 24
Figure S13: Extracted ion chromatogram of halogenated azaphilones from scaffold D' (H, Cl, Br and I) with their isotopic patterns. 25
Figure S14: Extracted ion chromatogram of halogenated azaphilones from scaffold E' (H, Cl, Br and I) with their isotopic patterns. 25
Figure S15: HRMS of compound **22.** 26
Figure S16: ^{1}H NMR spectrum (DMF-*d7*) of compound **22.** 29
Figure S17: ^{13}C NMR spectrum (DMF-*d7*) of compound **22.** 30
Figure S18: COSY NMR spectrum (DMF-*d7*) of compound **22.** 31
Figure S19: HSQC NMR spectrum (DMF-*d7*) of compound **22.** 32
Figure S20: HMBC NMR spectrum (DMF-*d7*) of compound **22.** 33
Figure S21: UV spectra in methanol of isolated azaphilone **22** 34

Table S1: Dereplicated ilicicolins from *N. discophora* crude extract. 3
Table S2: Dereplicated azaphilones from *P. sclerotiorum* crude extract. 7
Table S3: Basic genomes statistics. 13
Table S4: BUSCO score. 13
Table S5: *Ndi_Ili* biosynthetic gene cluster annotation. 14
Table S6: *Ndi_WSC72* biosynthetic gene cluster annotation. 15
Table S7: Annotated ilicicolins from *N. discophora* crude extract, synthetized by *Ndi_Ili* biosynthetic pathway. 17
Table S8: *Psc_Aza* biosynthetic gene cluster annotation. 18
Table S9: Annotated azaphilones from *P. sclerotiorum* crude extract and fractions, synthetized by *Psc_Aza* biosynthetic pathway. 22
Table S10: Comparative ^{1}H NMR spectroscopic data for compounds **22** (isolated during this study), sclerotiorin (**4**) sclerotioramine (**5**) and 5-chloroisorotiorin (**6a**) isolated by our group (reference 20 of the manuscript) and **6b** and **15** (from literature). 27
Table S11: Comparative ^{13}C NMR spectroscopic data for compounds **22** (isolated during this study), sclerotiorin (**4**) and sclerotioramine (**5**) isolated by our group (reference 20 of the manuscript) and **6b** and **15** (from literature). 28

Table S1: Dereplicated ilicicolins from *N. discophora* crude extract.

CN63								
$[M+H]^+$	Error (ppm)	RT	Formula	Name	Database	Cosine score	Annotation*	Ref
357.2422	0.6	14.9	$C_{23}H_{32}O_3$	LL-Z 1272β	GNPS	0.91	Level 2	[6]
363.2080	1.5	8.6	$C_{22}H_{35}ClO_4$	Ilicicolinol-2H_2O	In-House	0.92	Level 1	[7]
373.2364	2.5	16.5	$C_{23}H_{32}O_4$	Ilicicolinic acid B	In-House	0.93	Level 1	[8]
373.2373	0.0	13.2	$C_{23}H_{32}O_4$	Ilicicolinal G	In-House	0.85	Level 1	[7]
389.1867	2.8	14.7	$C_{23}H_{31}ClO_4$	Ilicicolinal H-H_2O	In-House	0.92	Level 1	[7]
389.1873	1.3	8.5	$C_{23}H_{35}ClO_6$	Ilicicolinic acid E-3H_2O	In-House	0.93	Level 1	[7]
391.2028	1.7	15.9	$C_{23}H_{31}ClO_3$	LL-Z1272α	MS-DIAL	0.89	Level 2	[9]
391.2456	5.9	11.7	$C_{23}H_{34}O_5$	Ilicicolinal I	In-House	0.82	Level 1	[7]
407.1951	8.0	17.0	$C_{23}H_{31}ClO_4$	LL-Z 1272δ	MS-DIAL	0.69	Level 2	[10]
407.1985	-0.4	14.2	$C_{23}H_{31}ClO_4$	Ilicicolinic acid A	In-House	0.93	Level 1	[8]
421.1773	0.8	12.2	$C_{23}H_{29}ClO_5$	Ascofuranone	MS-DIAL	0.74	Level 2	[11]
425.2082	1.7	8.5	$C_{23}H_{33}ClO_5$	Ilicicolinic acid C	In-House	0.82	Level 1	[8]

*Level 0 corresponds annotations from isolated pure compounds; level 1 to annotations by comparison with standards; level 2 to putative annotations (e.g., MS/MS library comparison or tentative structure); level 3 to a chemical class assignment. RT = Retention Time

Figure S1: Dereplicated ilicicolins structures with their theoretical exact masses and experimental fragmentation spectrum comparison with databases (in red *m/z* of database, in blue experimental *m/z* obtained). * represents the amount of H_2O losses during ionization.

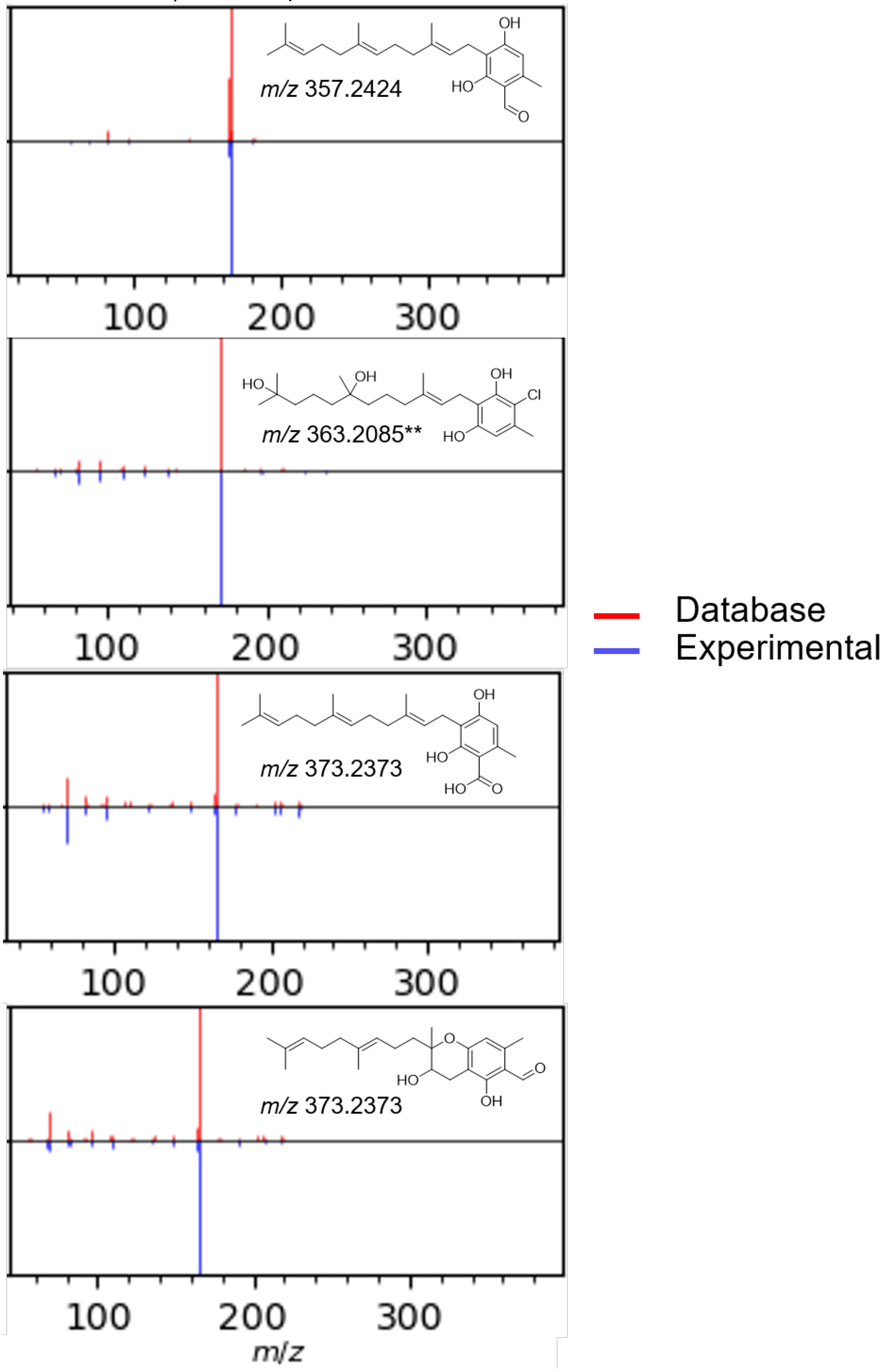

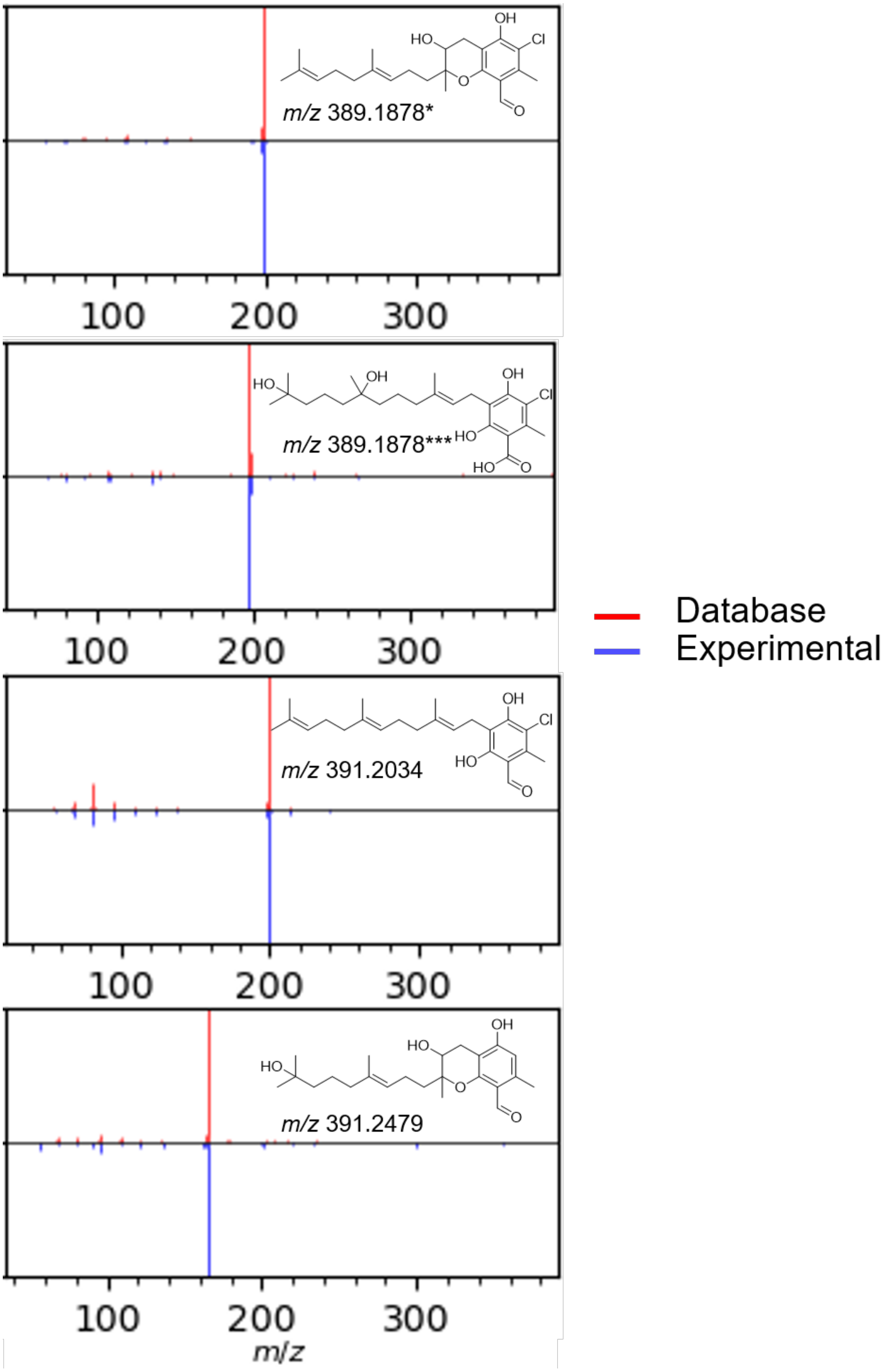

m/z 389.1878*
OH
HO
Cl
O
100
200
300
m/z 389.1878***
HO
OH
OH
Cl
HO
HO
O
100
200
300
m/z 391.2034
OH
Cl
HO
O
100
200
300
m/z 391.2479
OH
HO
HO
O
O
100
200
300
m/z
Database
Experimental

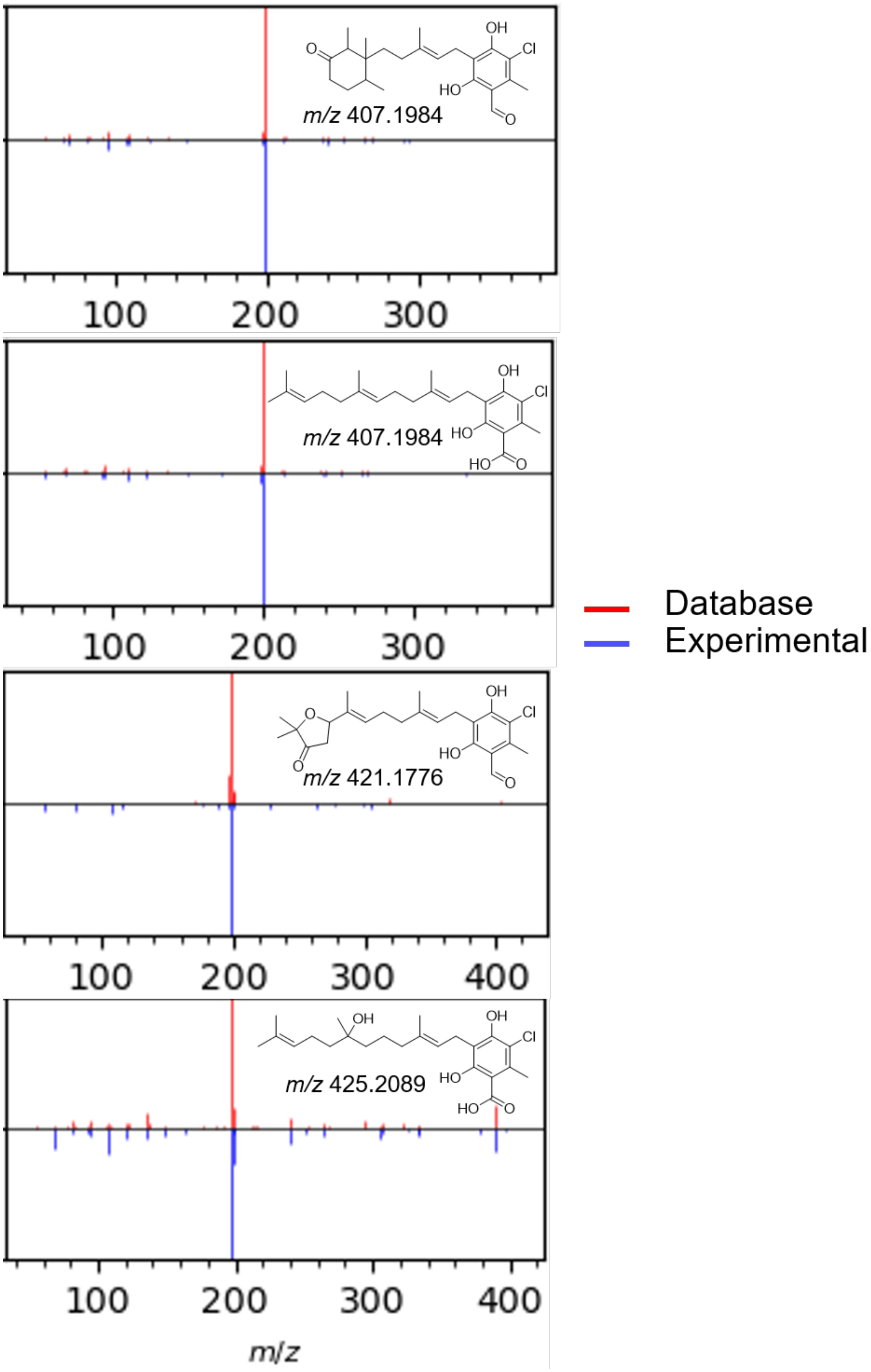

OH
Cl
O
HO
O
m/z 407.1984
100
200
300
OH
Cl
m/z 407.1984
HO
HO
O
100
200
300
OH
O
Cl
HO
O
m/z 421.1776
O
100
200
300
400
OH
OH
Cl
m/z 425.2089
HO
HO
O
100
200
300
400
m/z
Database
Experimental

Table S2: Dereplicated azaphilones from *P. sclerotiorum* crude extract.

CN111							
$[M+H]^+$	Error (ppm)	Formula	Name	Database	Cosine score	Annotation*	Ref
349.1202	-0.3	$C_{19}H_{21}ClO_4$		In-House	0.65	Level 2	[1]
356.1853	1.0	$C_{21}H_{25}NO_4$		In-House	0.86	Level 2	[1]
357.1700	-1.0	$C_{21}H_{24}O_5$		In-House	0.95	Level 2	[1]
359.1857	-1.1	$C_{21}H_{26}O_5$		In-House	0.74	Level 2	[1]
362.1522	-1.3	$C_{20}H_{24}ClNO_3$		In-House	0.88	Level 2	[1]
363.1353	1.3	$C_{20}H_{24}ClO_4$		In-House	0.85	Level 2	[1]
371.1844	2.4	$C_{22}H_{26}O_5$		In-House	0.84	Level 2	[1]
381.1697	-0.1	$C_{23}H_{24}O_5$	Isorotiorin	In-House	0.96	Level 2	[2]
385.2007	0.7	$C_{23}H_{28}O_5$		In-House	0.92	Level 2	[1]
390.1474	-1.9	$C_{21}H_{24}ClNO_4$	Sclerotioramine	MONA	0.90	Level 1	[1, 2]
391.1309	-0.6	$C_{21}H_{23}ClO_5$	Sclerotiorine	MS-DIAL	0.82	Level 1	[1, 2]
391.2114	0.3	$C_{22}H_{30}O_6$		In-House	0.86	Level 2	[1]
399.2169	-0.8	$C_{24}H_{30}O_5$		In-House	0.92	Level 2	[1]
404.1627	-1.0	$C_{22}H_{26}ClNO_4$		In-House	0.96	Level 2	[1]
405.1468	-1.2	$C_{22}H_{25}ClO_5$	Sclerketide B	In-House	0.95	Level 2	[1, 3]
407.1256	0.0	$C_{21}H_{23}ClO_6$		In-House	0.86	Level 2	[1]
415.1299	1.9	$C_{23}H_{23}ClO_5$	5-Chloroisorotiorin	In-House	0.93	Level 1	[1, 2]
417.1902	1.4	$C_{23}H_{28}O_7$	Geumsanol B	In-House	0.86	Level 2	[4]
419.1617	0.7	$C_{23}H_{27}ClO_5$		In-House	0.94	Level 2	[1]
433.1773	0.8	$C_{24}H_{29}ClO_5$		In-House	0.69	Level 2	[1]
434.1731	-0.5	$C_{23}H_{28}ClNO_5$	Isochromophilone VI	MSDIAL	0.83	Level 1	[1,5]
451.1526	1.3	$C_{23}H_{27}ClO_7$	Chlorogeumsanol B	In-House	0.84	Level 1	[1]
500.1839	-0.9	$C_{27}H_{30}ClNO_6$		In-House	0.96	Level 2	[1]

*Level 0 corresponds annotations from isolated pure compounds; level 1 to annotations by comparison with standards; level 2 to putative annotations (e.g., MS/MS library comparison or tentative structure); level 3 to a chemical class assignment.

Figure S2: Dereplicated azaphilone structures with their theoretical exact masses and experimental fragmentation spectrum comparison with databases.

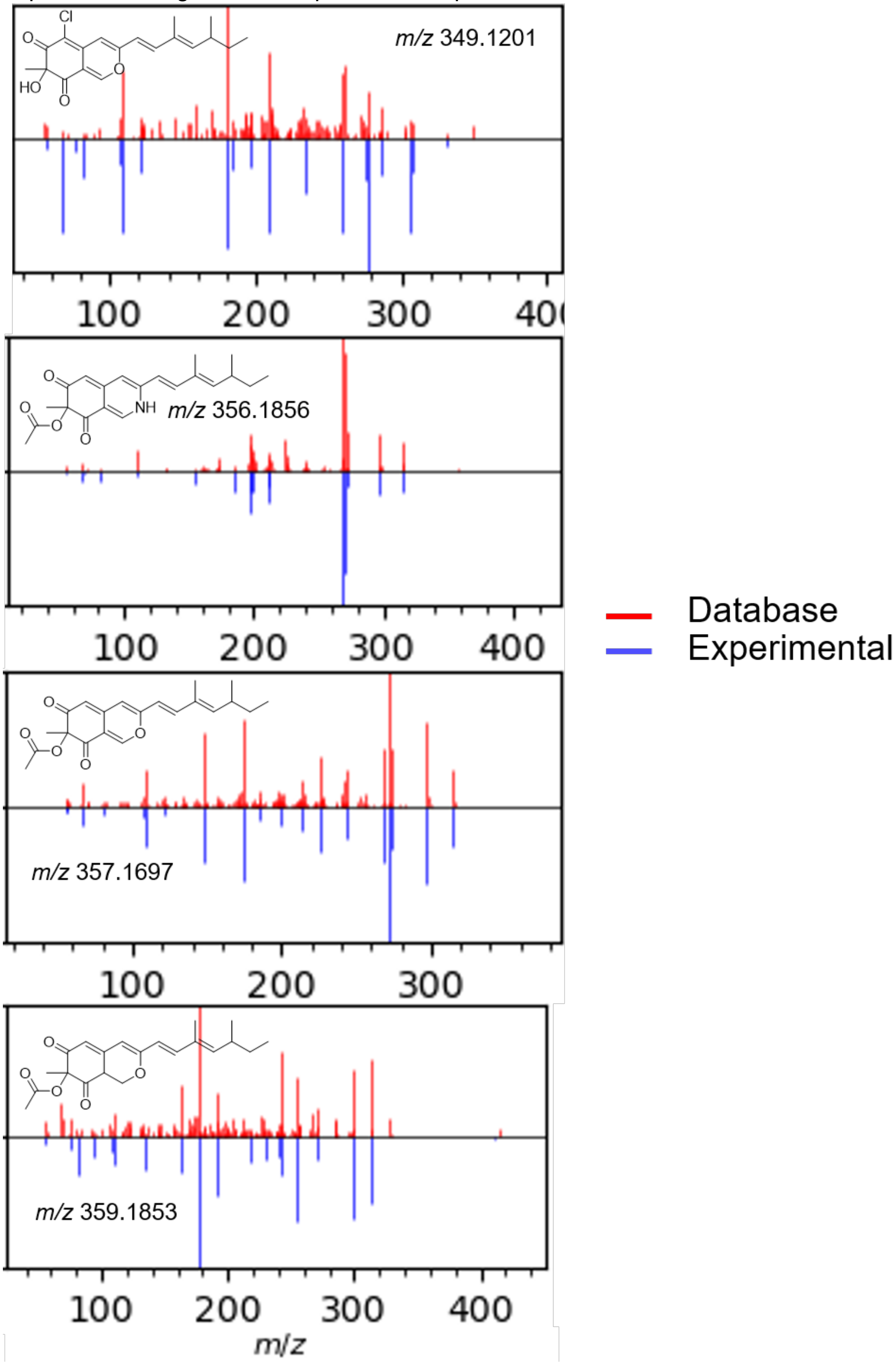

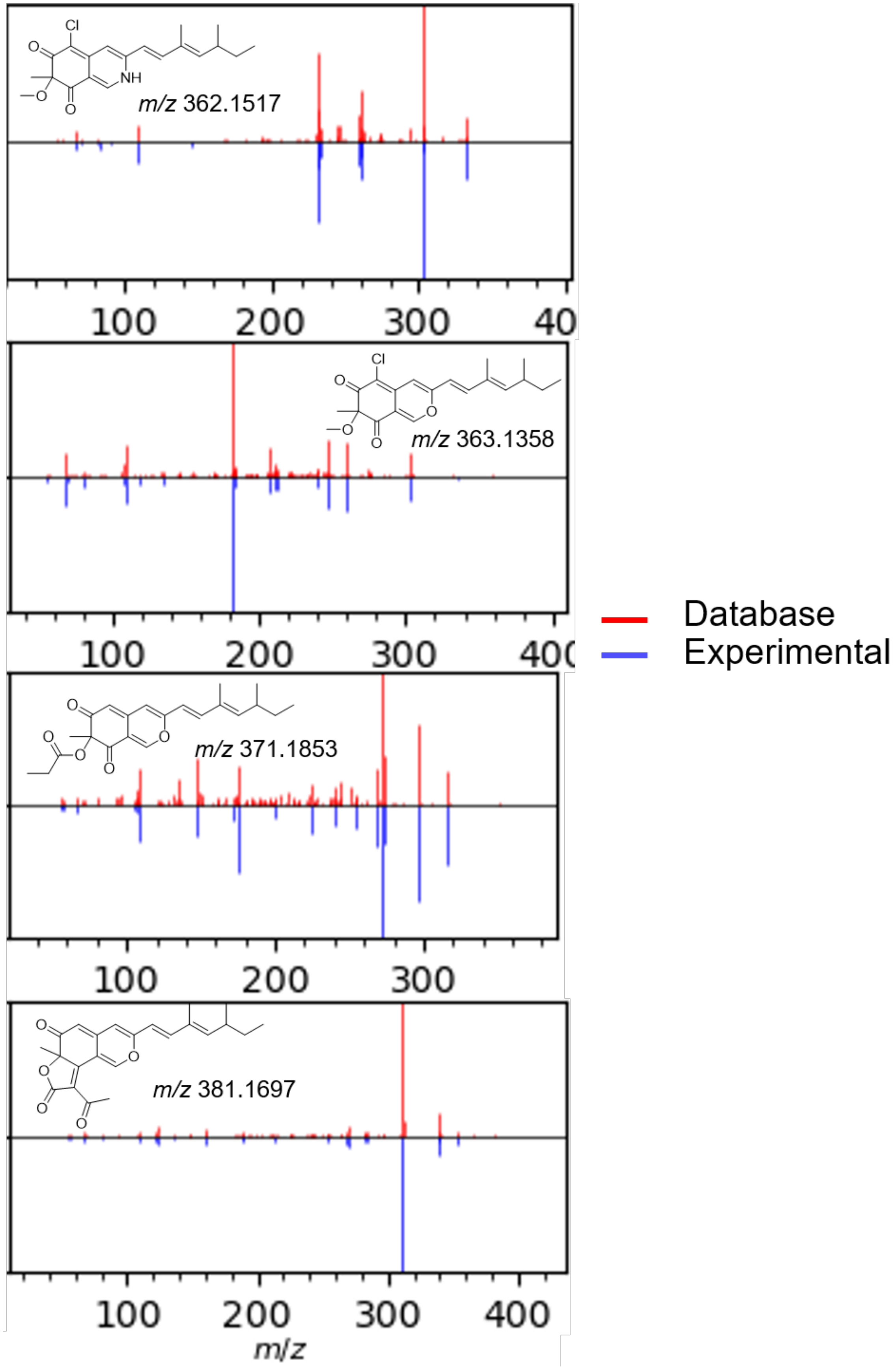

Cl
NH
m/z 362.1517
100
200
300
40
Cl
m/z 363.1358
100
200
300
400
m/z 371.1853
100
200
300
m/z 381.1697
100
200
300
400
m/z
Database
Experimental

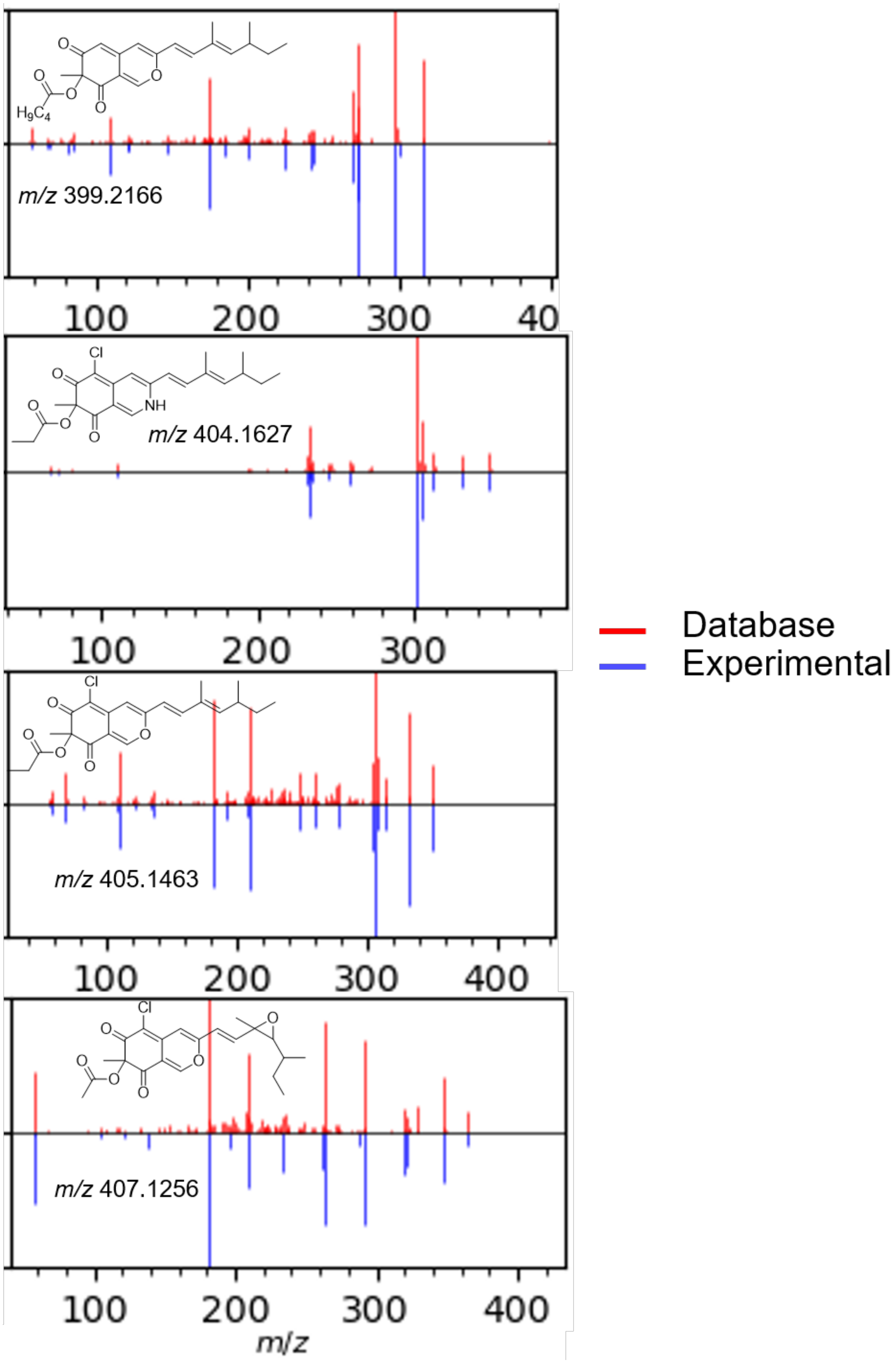

H9C4
m/z 399.2166
100
200
300
40
Cl
NH
m/z 404.1627
100
200
300
Cl
m/z 405.1463
100
200
300
400
Cl
m/z 407.1256
100
200
300
400
m/z
Database
Experimental

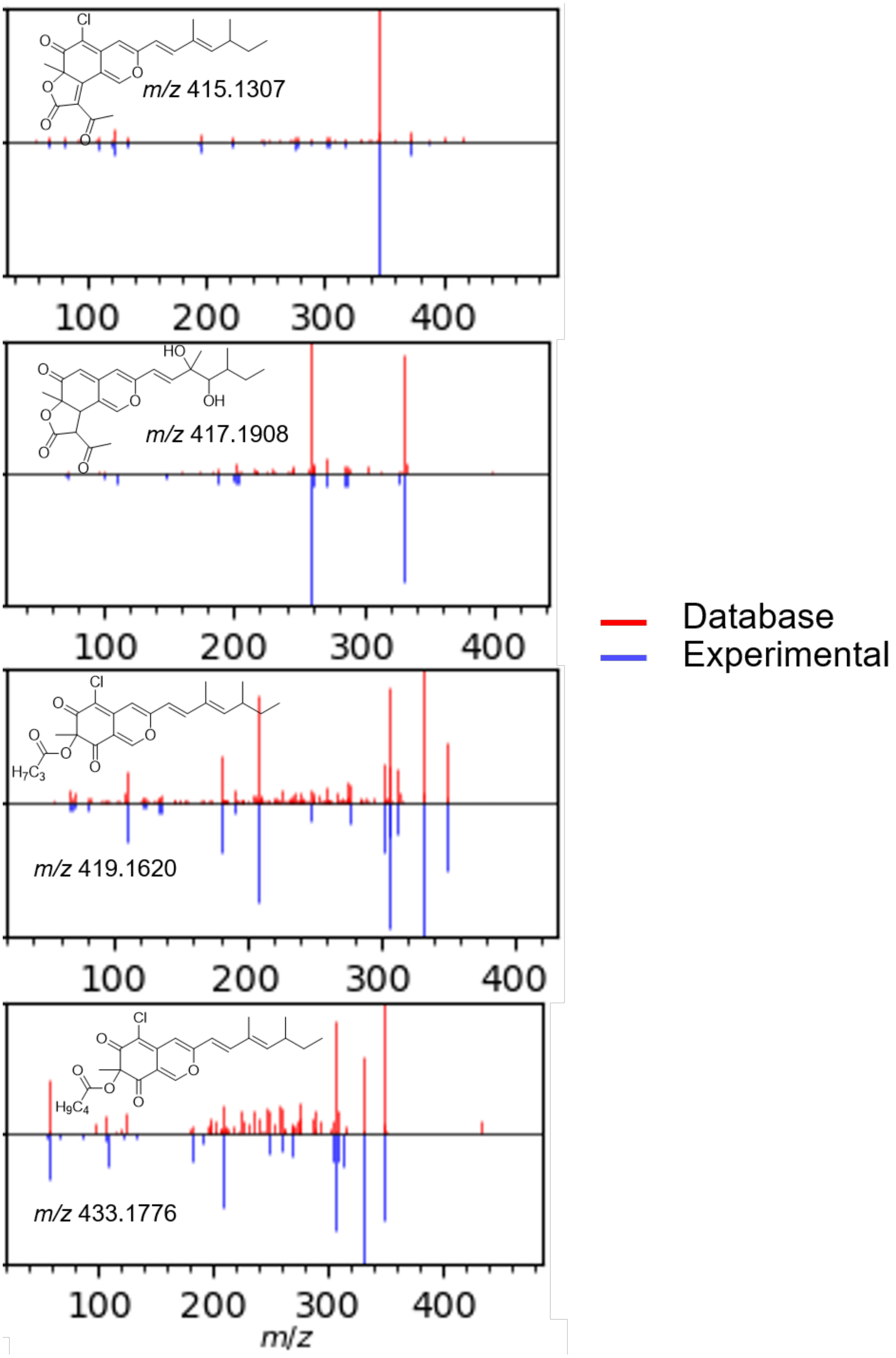

Cl
O
O
O
O
O
m/z 415.1307
100
200
300
400
HO
OH
m/z 417.1908
100
200
300
400
H7C3
m/z 419.1620
100
200
300
400
H9C4
m/z 433.1776
100
200
300
400
m/z
Database
Experimental

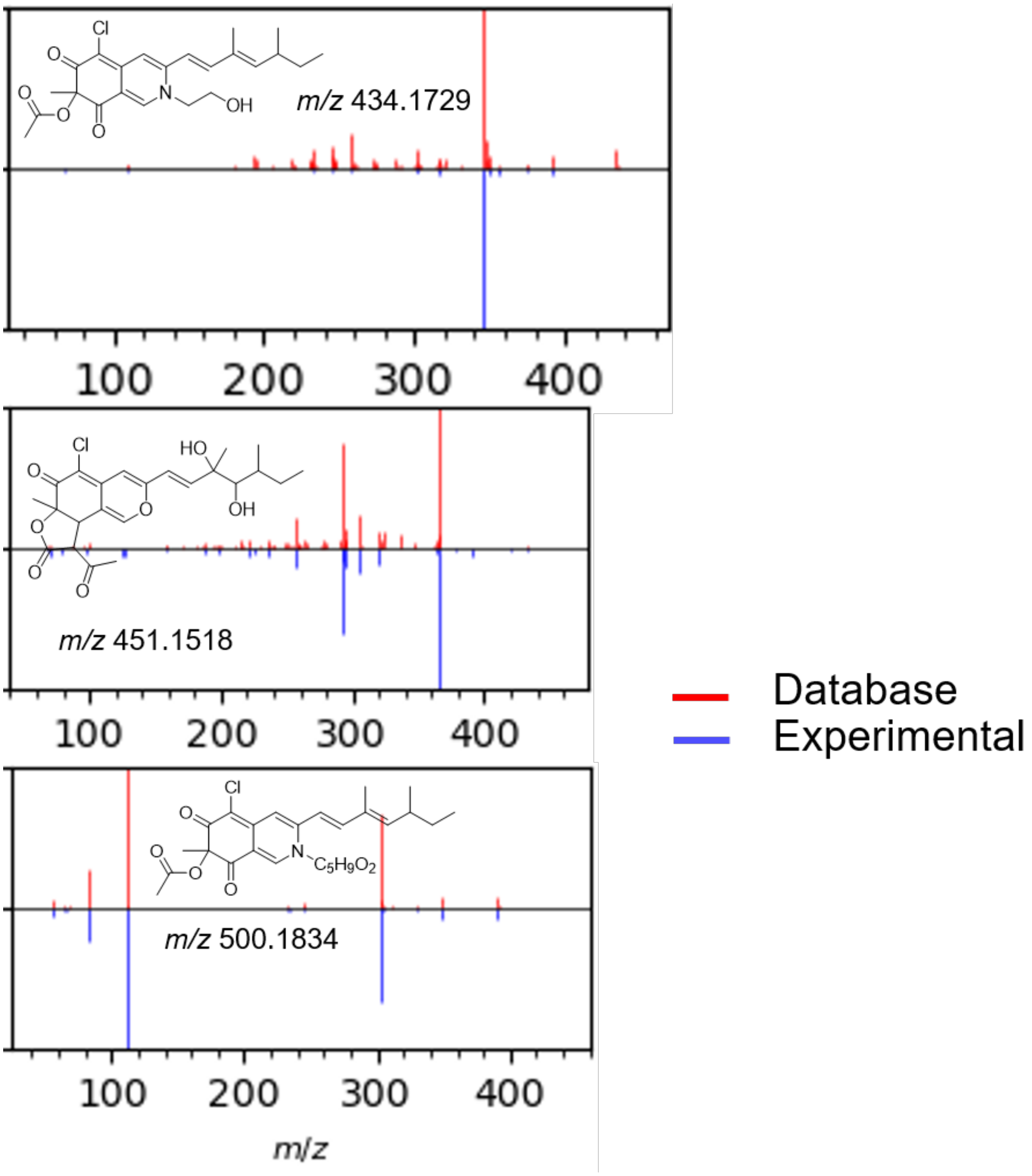

m/z 434.1729
m/z 451.1518
m/z 500.1834
m/z
Database
Experimental

Table S3: Basic genomes statistics.

	Genome size (Mpb)	Contigs	N50 (Mpb)	N75 (Mpb)	L50 (contigs)	L75 (contigs)	%GC
Neonectria discophora	41.6	26	4.03	3.02	3	8	54.2
Penicillium sclerotiorum	34.7	10	4.34	3.95	4	6	48.3

Table S4: BUSCO score.

Gene	Complete		Fragmented (%)	Missing (%)
	Single copy (%)	Duplicated (%)		
Neonectria discophora	99.3	0.0	0.0	0.7
Penicillium sclerotiorum	97.6	0.7	0.0	1.7

Table S5: *Ndi_Ili* biosynthetic gene cluster annotation.

Gene name	Size (AA)	Best match Swissprot	Global alignement (I/S/G) %	Global alignement (I/S/G) AA	domain	_ID	Plausible function	Duplicated
Ndi_Ili_A	2014	A0A455R5P9	56/70/8	1201/1506/179	beta-ketoacyl synthase<br>acyl transferase<br>product template<br>acyl carrier protein<br>thioesterase	PF00109.26<br>PF00698.21<br>TIGR04532<br>PF00550.25<br>IPR001031	Non reducing polyketide synthase	N
Ndi_Ili_B	297	A0A455R413	49/59/16	167/203/55	prenyltransferase	PF01040.18	Prenyltransferase	N
Ndi_Ili_C	1065	A0A455R7E6	60/75/4	662/826/40	AMP-binding,<br>NAD dependent epimerase/dehydratase	PF00501.28<br>PF01370.21	Non-canonical non-ribosomal peptide synthetase	N
Ndi_Ili_D	553	A0A455R7M0	68/79/5	387/447/31	tryptophan halogenase	PF04820.14	FAD dependant halogenase	N
Ndi_Ili_E	674	No reviewed protein			sugar (and other) transporter, major facilitator	PF00083.24<br>PF07690.16	Transporter	N
Ndi_Ili_F	451	No reviewed protein			ATPase family associated with various cellular activities	PF00004.29	ATPase	N
Ndi_Ili_G	532	No reviewed protein						N
Ndi_Ili_H	418	No reviewed protein						N
Ndi_Ili_I	442	No reviewed protein					unknown function	N
Ndi_Ili_J	782	No reviewed protein					unknown function	N

I: identity, S: similarity, G: gap

Table S6: *Ndi_WSC72* biosynthetic gene cluster annotation.

Gene name	Size (AA)	Best match Swissprot	Global alignement (I/S/G) %	Global alignement (I/S/G) AA	domain	_ID	Plausible function	Duplicated
Ndi_WSC72_A	1786	C5FM57	56/70/5	1030/1296/96	ketoacyl synthase<br>acyl transferase<br>product template<br>acyl carrier protein	PF00109.26<br>PF00698.21<br>TIGR04532.6<br>PF00550.25	Non reducing polyketide synthase	N
Ndi_WSC72_B	556	Q2UPC7	57/72/5	322/408/31	tryptophan halogenase	PF04820.14	FAD dependant halogenase	N
Ndi_WSC72_C	220	No reviewed protein			glutathione S-transferase	IPR040079	Glutathione S-transferase	N
Ndi_WSC72_D	337	G3KLH5	54/71/3	174/227/11	β-lactamase	PF00753	β-lactamase	N
Ndi_WSC72_E	378	Q9P900	39/58/4	150/227/14	O-methyltransferase	PF00891	methyltransferase	N
Ndi_WSC72_F	516	No reviewed protein			copper oxidase	PF00394<br>PF07731	unknown function	N
Ndi_WSC72_G	261	A0A179G9G5	37/44/45	164/194/198	zinc finger	PF00172	Transcription factor	N
Ndi_WSC72_H	427	No reviewed protein			monooxygenases	PF01494	monooxygenase	N
Ndi_WSC72_I	532	No reviewed protein					unknown function	N
Ndi_WSC72_J	413	No reviewed protein					unknown function	N
Ndi_WSC72_K	292	No reviewed protein					unknown function	N
Ndi_WSC72_L	918	No reviewed protein					unknown function	N

Figure S3: Cluster comparison of azaphilone biosynthetic gene clusters described in literature with the putative azaphilone biosynthetic gene cluster of *Penicillium sclerotiorum* SNB-CN111. Only functions of tailoring enzyme related to *P. sclerotiorum* are annotated. BGC identifier are accession number from MIBIG repository. Clinker Software (https://github.com/gamcil/clinker).

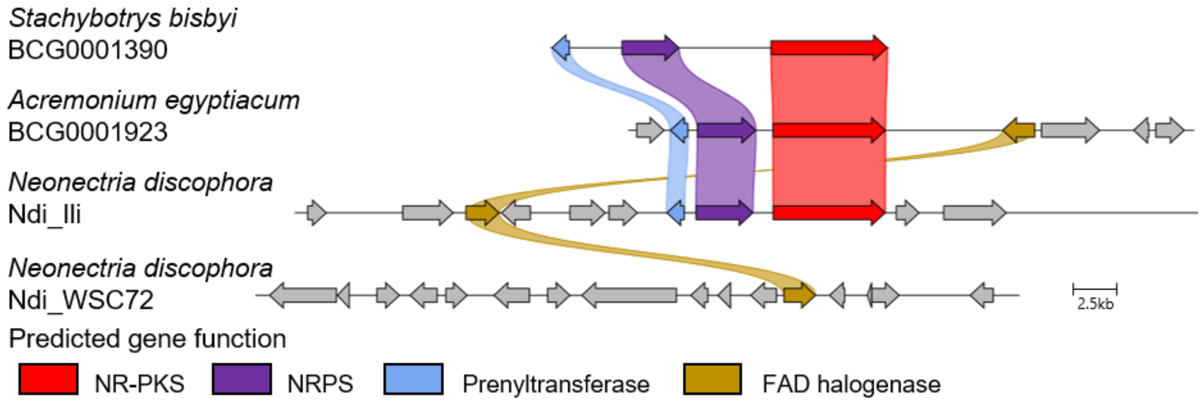

Table S7: Annotated ilicicolins from *N. discophora* crude extract, synthetized by *Ndi_Ili* biosynthetic pathway.

CN63					
**ID**	**$[M+H]^+$**	**Error (ppm)**	**Formula**	**Identification**	**Ref**
**9**	373.2364	2.5	$C_{23}H_{32}O_4$	Dereplication, standard	[8]
**2**	407.1985	1.1	$C_{23}H_{31}ClO_4$	Dereplication, standard	[8]
**8**	357.2422	0.6	$C_{23}H_{32}O_3$	Dereplication, *in silico*	[6]
**1**	391.2028	1.7	$C_{23}H_{31}ClO_3$	Dereplication, *in silico*	[9]

Table S8: *Psc_Aza* biosynthetic gene cluster annotation.

Gene name	Size (AA)	Best match Swissprot	Global alignement (I/S/G) %	Global alignement (I/S/G) AA	Pfam domain	PFAM_ID	Plausible_function	Duplicated
Psc_Aza_A	2584	Q0CF75	65/77/7	1744/2076/185	beta-ketoacyl synthase<br>acyl transferase<br>polyketide synthase dehydratase<br>methyltransferase<br>enoylreductase<br>ketoreductase<br>phosphopantetheine attachment site	PF00109.26<br>PF00698.21<br>PF14765.6<br>PF08242.12<br>IPR020843<br>PF00107.26<br>PF08659.10	Highly reducing polyketide synthase	N
Psc_Aza_B	2726	A0A0K0MCJ4	67/78/5	1870/2188/129	beta-ketoacyl synthase<br>acyl transferase<br>phosphopantetheine attachment site<br>methyltransferase<br>terminal domain	PF16073.5<br>PF00109.26<br>PF00698.21<br>PF00550.25<br>IPR013120	Non reducing polyketide synthase	N
Psc_Aza_C	352	Q0CF72	44/55/32	208/256/152	FAD-binding domain_3	PF01494.19	Monooxygenase FAD dependant	N
Psc_Aza_D	449	A0A084B9Z3	34/50/8	162/239/40	transferase	PF02458	Acyltransferase	N
Psc_Aza_E	471	Q0CF74	49/66/4	232/318/18	FAD binding domain	PF01565.23	FAD-linked oxidoreductase	N
Psc_Aza_F	341	No reviewed protein			zinc-binding dehydrogenase,<br>alcohol dehydrogenase GroES-like	PF00107.26<br>PF08240.12	Dehydrogenase	N
Psc_Aza_G	385	No reviewed protein			alcohol dehydrogenase GroES-like	PF08240.12	Alcohol dehydrogenase	N
Psc_Aza_H	561	Q0CCX4	55/68/8	318/392/43	tryptophan halogenase	PF04820.14	FAD-dependant halogenase	N
Psc_Aza_I	590	No reviewed protein					Unknow function	N
Psc_Aza_J	227	No reviewed protein			amine oxidoreductase	PF01593.24	Amine oxidase	N
Psc_Aza_K	306	No reviewed protein			G protein-coupled glucose receptor	PF11970.8	G protein-coupled receptor	N
Psc_Aza_L	590	No reviewed protein			major facilitator	PF07690.16	Efflux pump	N
Psc_Aza_M	407	No reviewed protein					Unknow function	N

I: identity, S: similarity, G: gap

Figure S4: Cluster comparison of azaphilone biosynthetic gene clusters described in literature with the putative azaphilone biosynthetic gene cluster of *Penicillium sclerotiorum* SNB-CN111. Only functions of tailoring enzyme related to *P. sclerotiorum* are annotated. BGC identifier are accession number from MIBIG repository. Clinker Software (https://github.com/gamcil/clinker).

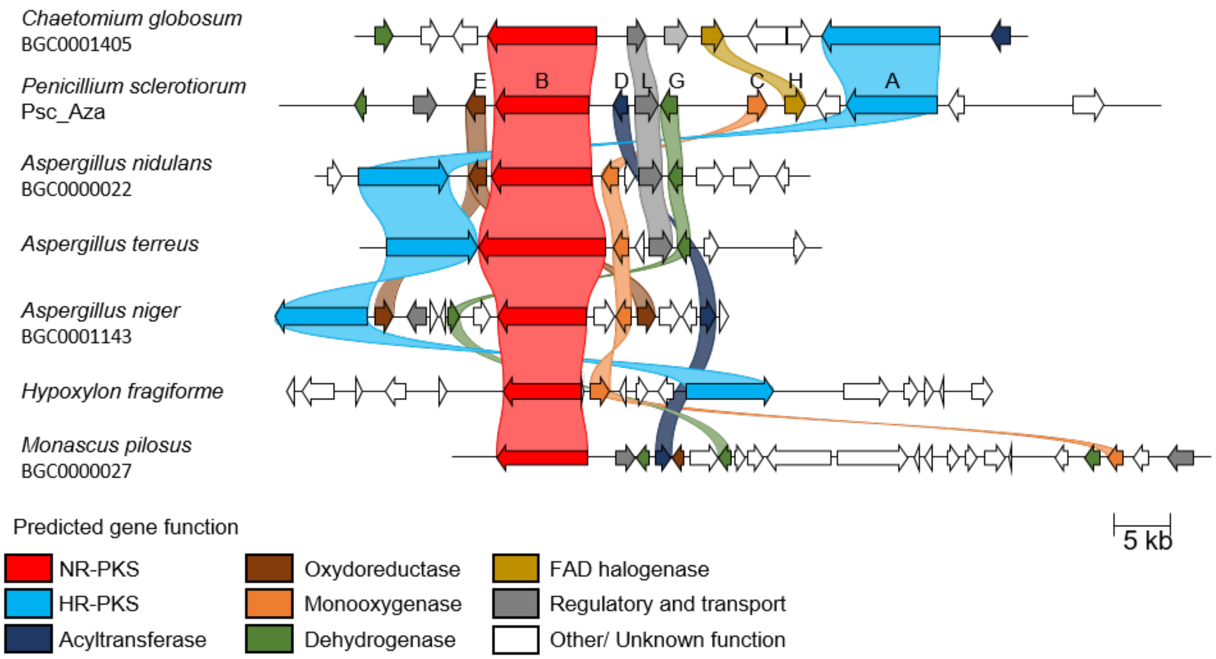

Figure S5: MS/MS spectra of [**10**+H]$^+$ and the corresponding fragmentation scheme.

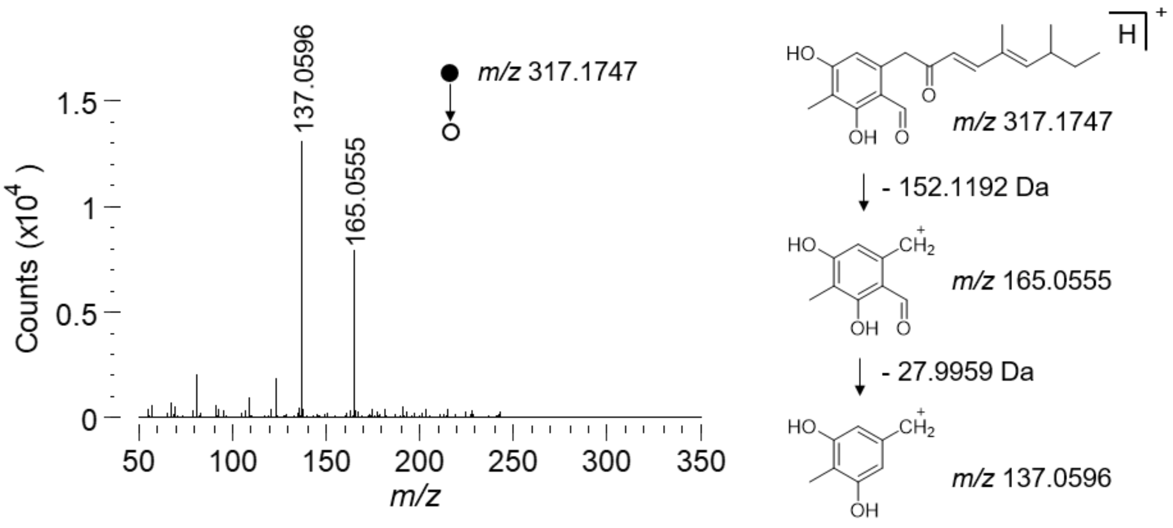

Figure S6: MS/MS spectra of [**11**+H]$^+$ and the corresponding fragmentation scheme.

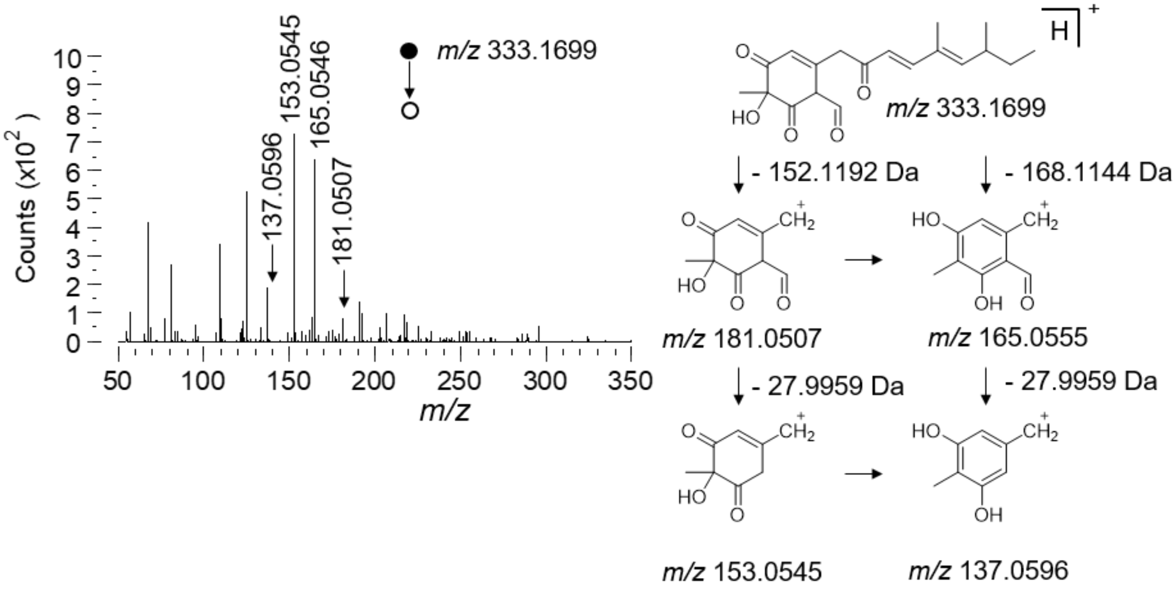

Figure S7: MS/MS spectra of [**12**+H]$^+$ and the corresponding fragmentation scheme.

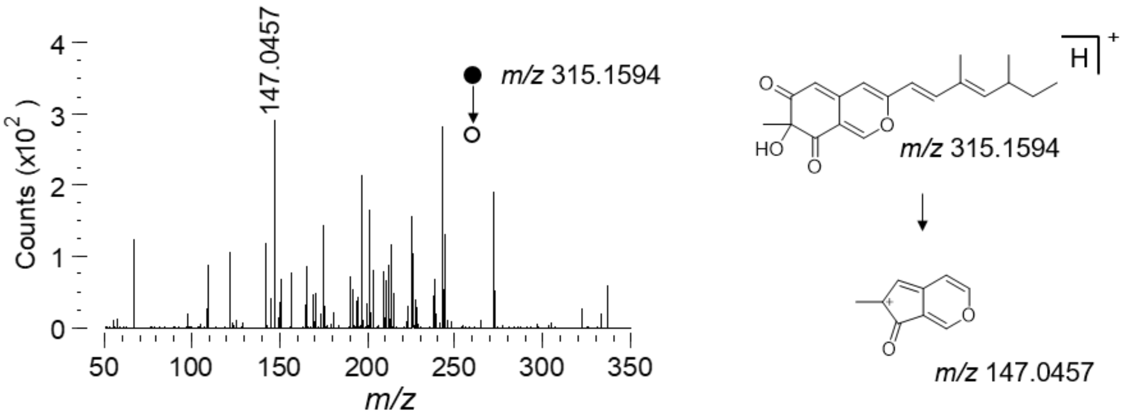

Figure S8: Comparison of intermediates **12** and **21** fragmentation spectra with their common neutral losses corresponding to H/Cl differences.

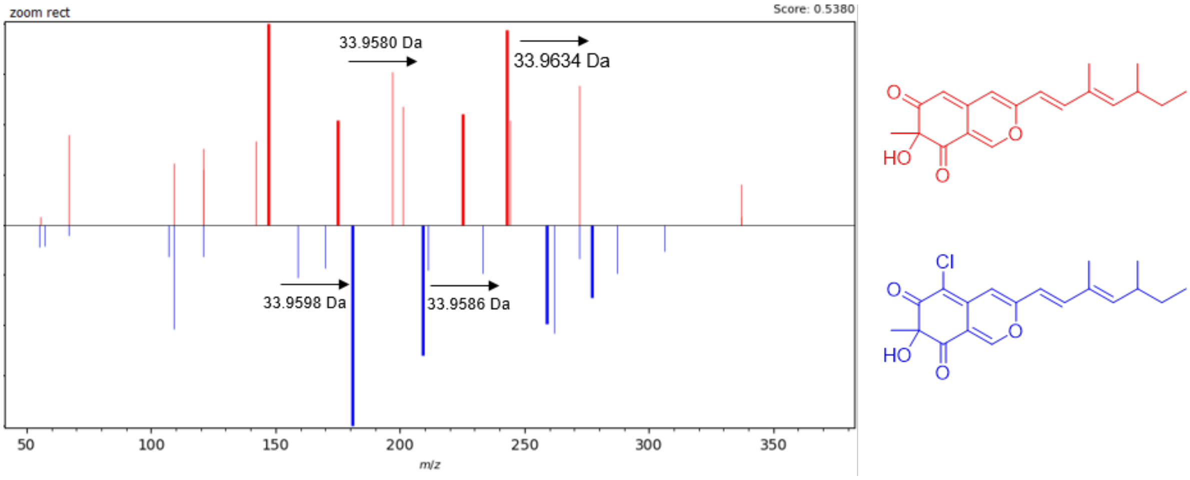

Table S9: Annotated azaphilones from *P. sclerotiorum* crude extract and fractions, synthetized by *Psc_Aza* biosynthetic pathway.

CN111					
**ID**	**[M+H]$^+$**	**Error (ppm)**	**Formula**	**Identification**	**Ref**
**10**	317.1747	0.1	$C_{19}H_{24}O_4$	Manual annotation, *in silico*	This study
**11**	333.1699	-0.8	$C_{19}H_{24}O_5$	Manual annotation, *in silico*	This study
**12**	315.1594	-1	$C_{19}H_{22}O_4$	Manual annotation, *in silico*	[12]
**21**	349.1205	-1.1	$C_{19}H_{21}ClO_4$	Dereplication, *in silico*	[1]
**13**	357.1705	-2.4	$C_{21}H_{24}O_5$	Dereplication, *in silico*	[1]
**4**	391.1309	-0.6	$C_{21}H_{23}ClO_5$	Dereplication, standard	[1, 2]
**19**	361.2021	-3.2	$C_{21}H_{28}O_5$	Manual annotation, *in silico*	This study
**20**	395.1626	-1.6	$C_{21}H_{27}Cl_{O5}$	Dereplication, *in silico*	[1, 2]
**15**	381.1691	1.5	$C_{23}H_{24}O_5$	Dereplication, *in silico*	[1, 2]
**6**	415.1312	-1.3	$C_{23}H_{23}ClO_5$	Dereplication, *standard*	[1, 2]
**16**	383.1859	-1.6	$C_{23}H_{26}O_5$	Dereplication, *in silico*	[1, 2]
**17**	417.1467	-0.9	$C_{23}H_{25}ClO_5$	Dereplication, *in silico*	[1, 2]

Figure S9: Extracted ion chromatogram of halogenated ilicicolins from scaffold A (H, Cl and Br) with their isotopic patterns.

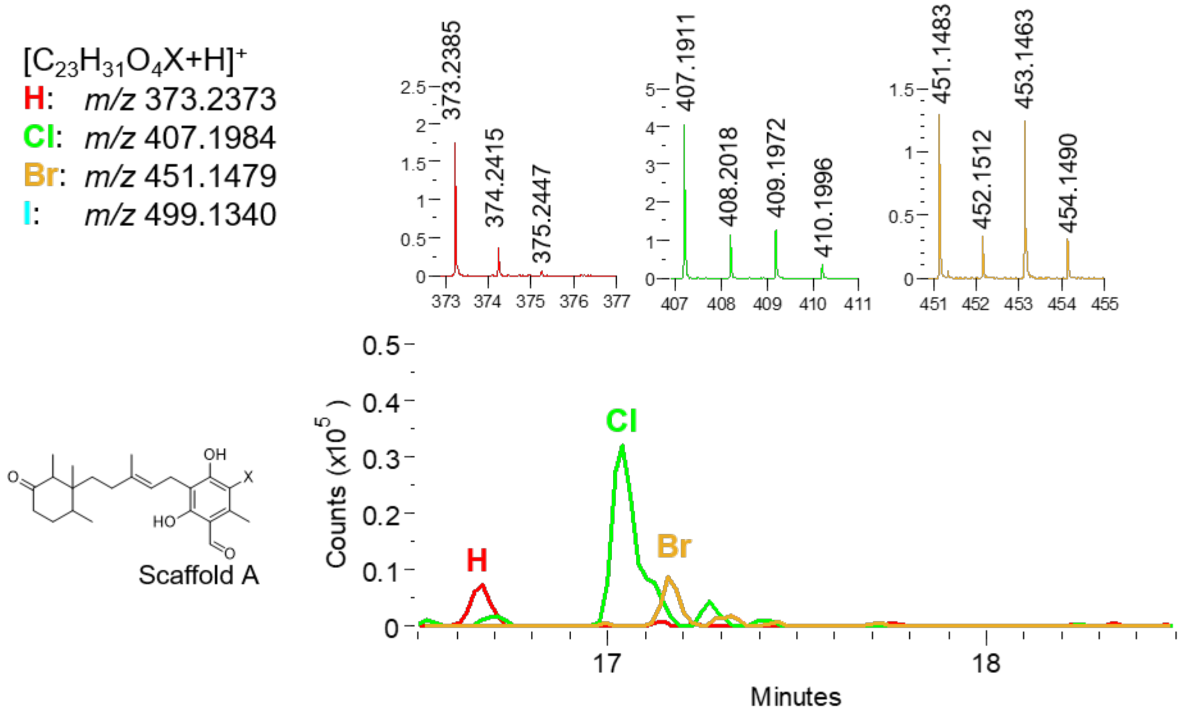

Figure S10: Extracted ion chromatogram of halogenated ilicicolins from scaffold B (H, Cl and Br) with their isotopic patterns.

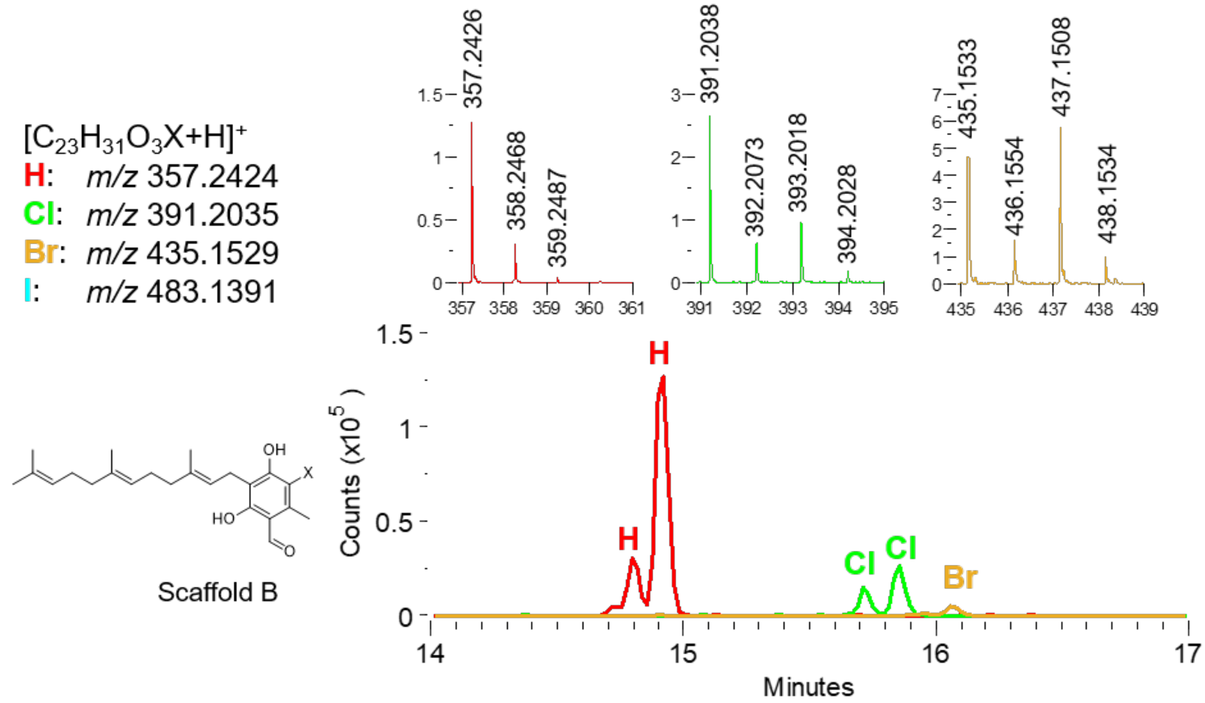

Figure S11: Extracted ion chromatogram of halogenated azaphilones from scaffold B' (H, Cl, Br and I) with their isotopic patterns.

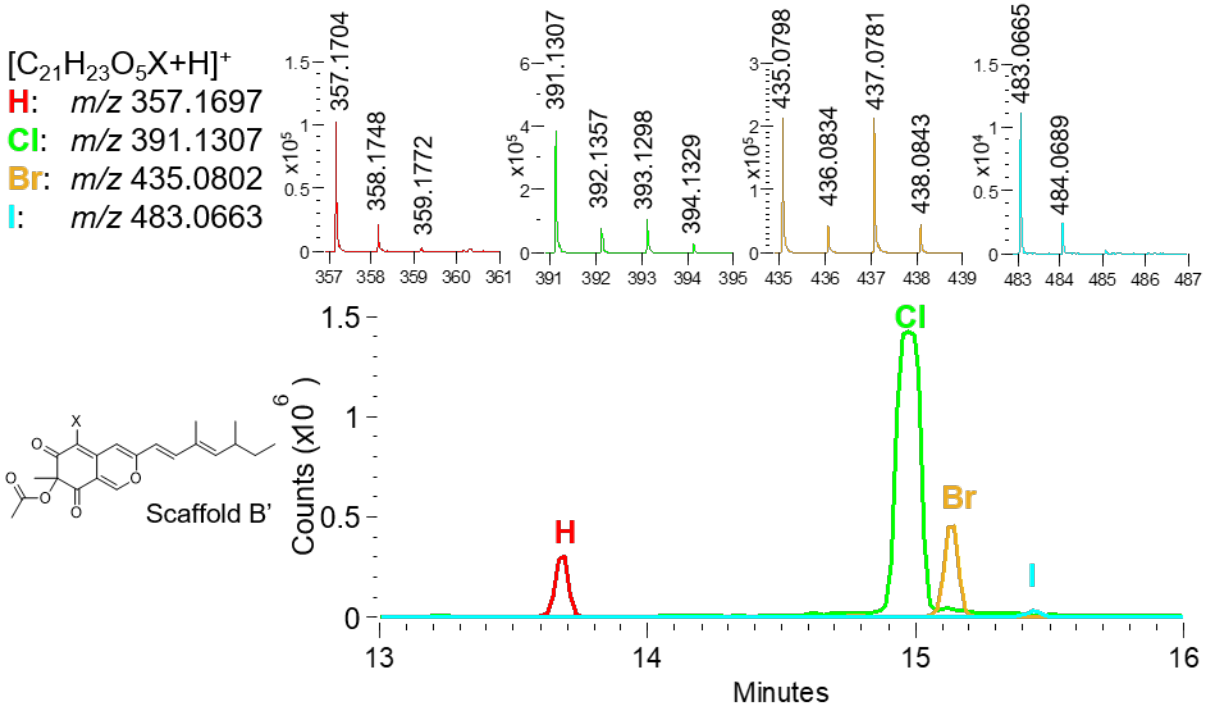

Figure S12: Extracted ion chromatogram of halogenated azaphilones from scaffold C' (H, Cl, Br and I) with their isotopic patterns.

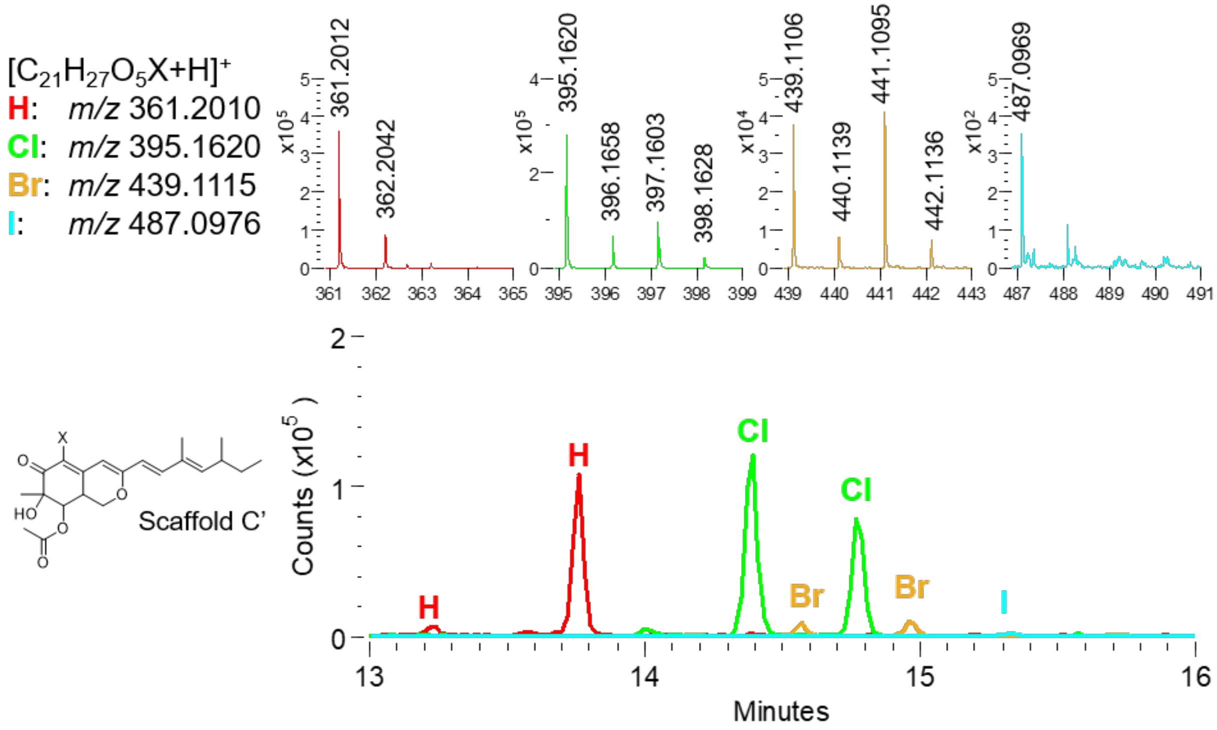

Figure S13: Extracted ion chromatogram of halogenated azaphilones from scaffold D' (H, Cl, Br and I) with their isotopic patterns.

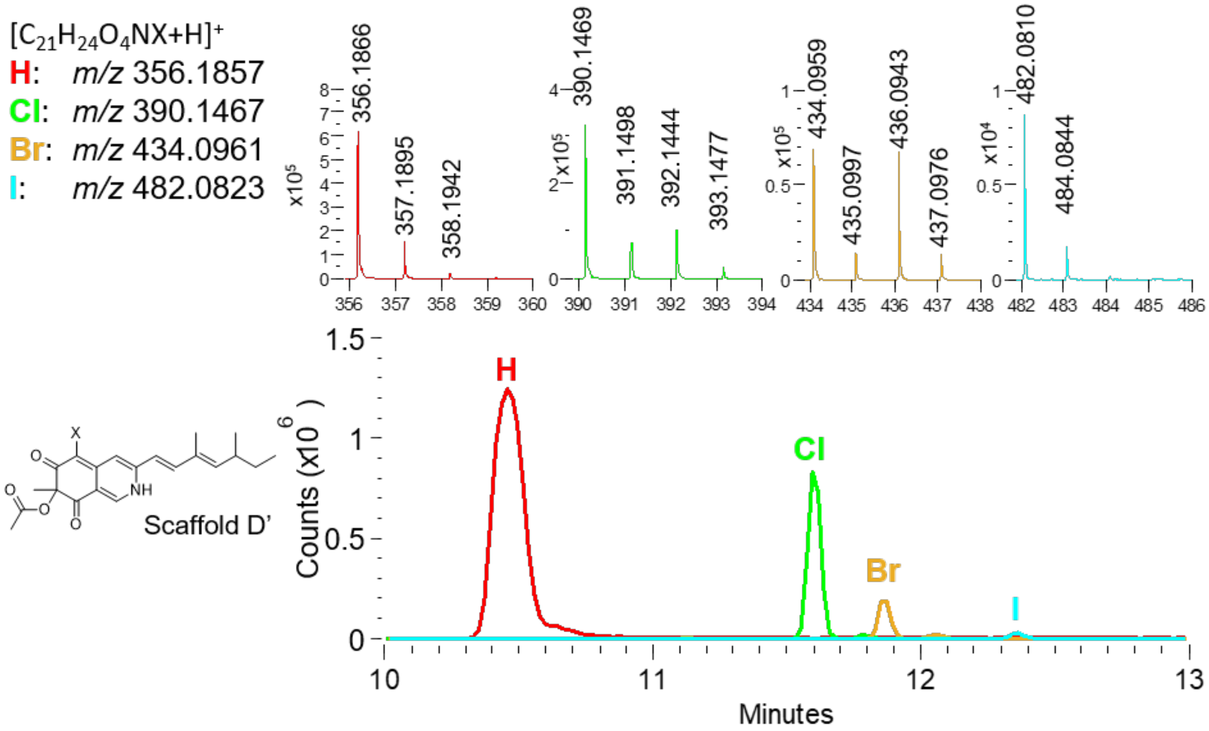

Figure S14: Extracted ion chromatogram of halogenated azaphilones from scaffold E' (H, Cl, Br and I) with their isotopic patterns.

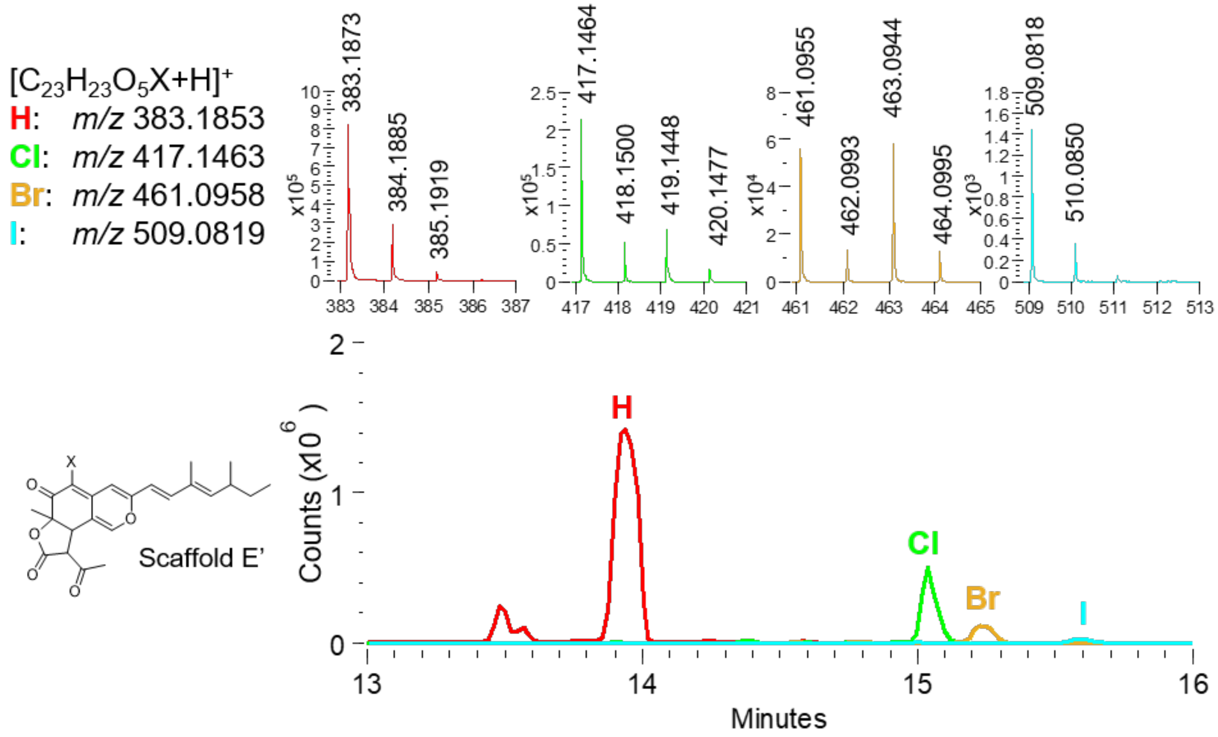

Figure S15: HRMS of compound **22.**

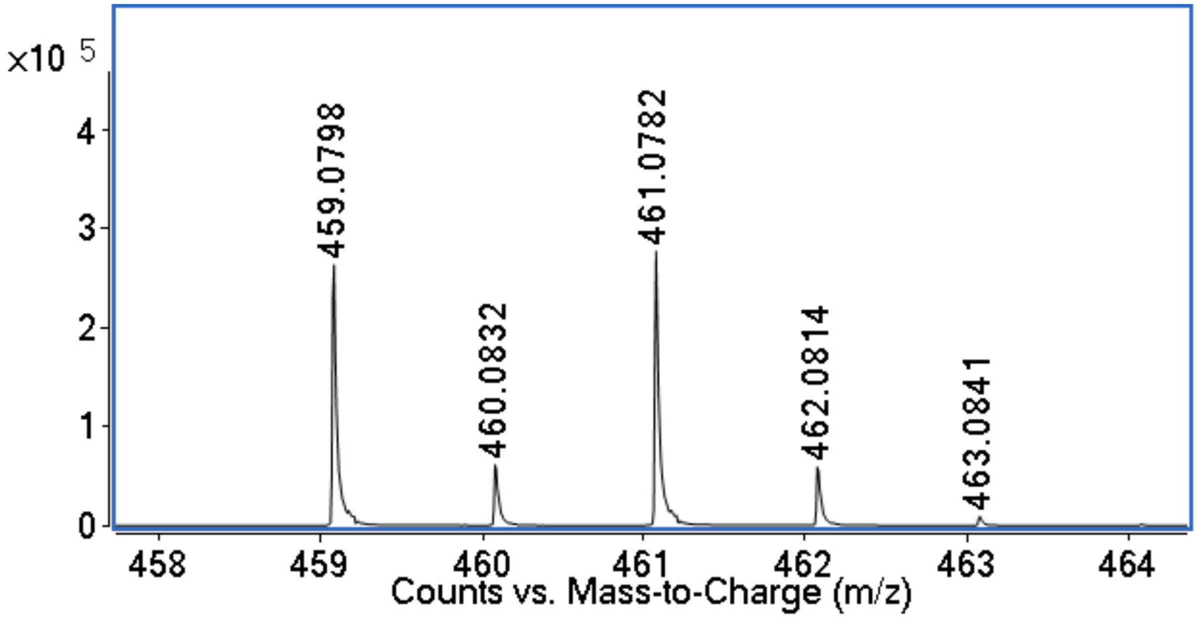

Table S10: Comparative ^{1}H NMR spectroscopic data for compounds **22** (isolated during this study), sclerotiorin (**4**) sclerotioramine (**5**) and 5-chloroisorotiorin (**6a**) isolated by our group (reference 20 of the manuscript) and **6b** and **15** (from literature)

Position	**22**	**4**	**5**	**6a**	**6b**	**15**
	DMF-*d7*[a]	$CDCl_3$[b]	$CDCl_3$[b]	MeOD[c]	$CDCl_3$[b]	MeOD[c]
1	8.82, s	7.90, s	7.93, s	8.88, s	8.84, s	8.88, s
4	6.98, s	6.61, s	6.86, s	6.81, s	6.16, s	6.81, s
5					6.34, s	
9	6.78, d(16.1)	6.51, d(15.9)	6.13, d(16.1)	6.35, d(15.6)	5.97, d(15.8)	6.35, d(15.6)
10	7.26, d(16.2)	7.03, d(15.9)	7.04, d(16.7)	7.19, (16.0)	7.04, d(15.8)	7.19, d(16.0)
12	5.87, d(10.2)	5.67, d(10.0)	5.69, d(9.9)	5.77, d(9.8)	5.67, d(9.9)	5.77, d(9.8)
13	2.55, m	2.45, m	2.47, m	2.53,m	2.48, m	2.52, m
14	1.45/1.34, m	1.29/1.39, m	1.30/1.40, m	1.34/1.46, m	1.35, m	1.46/1.34, m
15	0.87, t(7.4)	0.83, t(7.5)	0.84, t(7.5)	0.89, t(7.6)	0.85, t(7.5)	0.89, t(7.6)
16	1.02, d(6.7)	0.98, d(6.8)	0.99, d(6.8)	1.03, d(6.6)	1.00, d(6.7)	1.03, d(6.6)
17	1.92, s	1.81, s	1.83, s	1.88, s	1.81, s	1.88, s
18	1.72, s	1.53, s	1.57, s	1.68, s	1.69, s	1.68, s
20		2.13, s	2.16, s			
5''	2.57, s			2.55, s	2.59, s	2.55, s

[a] DMF-*d7*, 700 MHz, this study
[b] $CDCl_3$, no indication[13]
[c] MeOD, 500 MHz[1]

Table S11: Comparative ^{13}C NMR spectroscopic data for compounds **22** (isolated during this study), sclerotiorin (**4**) and sclerotioramine (**5**) isolated by our group (reference 20 of the manuscript) and **6b** and **15** (from literature)

	**22**	**4**	**5**	**6b**	**15**
	DMF-*d7*[a]	$CDCl_3$[b]	$CDCl_3$[b]	$CDCl_3$[b]	$CDCl_3$[c]
1	153.2 CH	152.8 CH	138.4 CH	153.1, CH	151.9, CH
3	159.7, C	158.4, C	146.3, C	156.3, C	158.0, C
4	109.5, CH	110.8, CH	110.3, CH	108.2, CH	105.5, CH
4a	143.4, C	138.8, C	147.1, C	123.1, C	139.4, C
5	100.8, C	106.6, C	101.5, C	105.3, CH	108.7, C*
6	184.5, C	186.0, C	183.7, C	189.9, C	194.1, C
7	88.4, C	84.7, C	85.4, C	87.5, C	87.4, C
8	164.2, C	192.0, C	193.3, C	165.6, C	163.6, C
8a	112.1, C	114.8, C	114.2, C	110.7, C	110.2, C*
9	118.1, CH	115.9, CH	116.4, CH	115.4, CH	115.6, CH
10	143.6, CH	143.0, CH	142.9, CH	144.1, CH	143.5, CH
11	133.9, C	132.2, C	132.0, C	131.8, C	131.9, C
12	149.1, CH	149.0, CH	148.7, CH	148.3, CH	149.2, CH
13	35.9, CH	35.3, CH	35.1, CH	35.5, CH	35.1, CH
14	30.3, CH_2	30.2, CH_2	30.6, CH_2	30.0, CH_2	29.8, CH_2
15	12.5, CH_3	12.1, CH_3	12.0, CH_3	11.9, CH_3	11.8, CH_3
16	20.8, CH_3	20.2, CH_3	20.1, CH_3	20.1, CH_3	20.0, CH_3
17	12.9, CH_3	12.5, CH_3	12.4, CH_3	12.4, CH_3	12.2, CH_3
18	26.4, CH_3	22.7, CH_3	23.6, CH_3	26.4, CH_3	26.1, CH_3
19		170.3, C	170.9, C		
20		20.3, CH_3	20.6, CH_3		
2'	169.4, C			168.3, C	167.7, C
3'	124.8, C			142.3, C	123.7, C
4'	195.2, C			194.4, C	182.9, C
5'	30.5, CH_3			30.0, CH_3	29.9, CH_3

[a] DMF-*d7*, 700 MHz, this study.
[b] $CDCl_3$, no indication[13]
[c] $CDCl_3$, 100 MHz[14]
*Authors indicate that these assignments were interchangeable.

Figure S16: ^{1}H NMR spectrum (700 MHz, DMF-*d7*) of compound **22.**

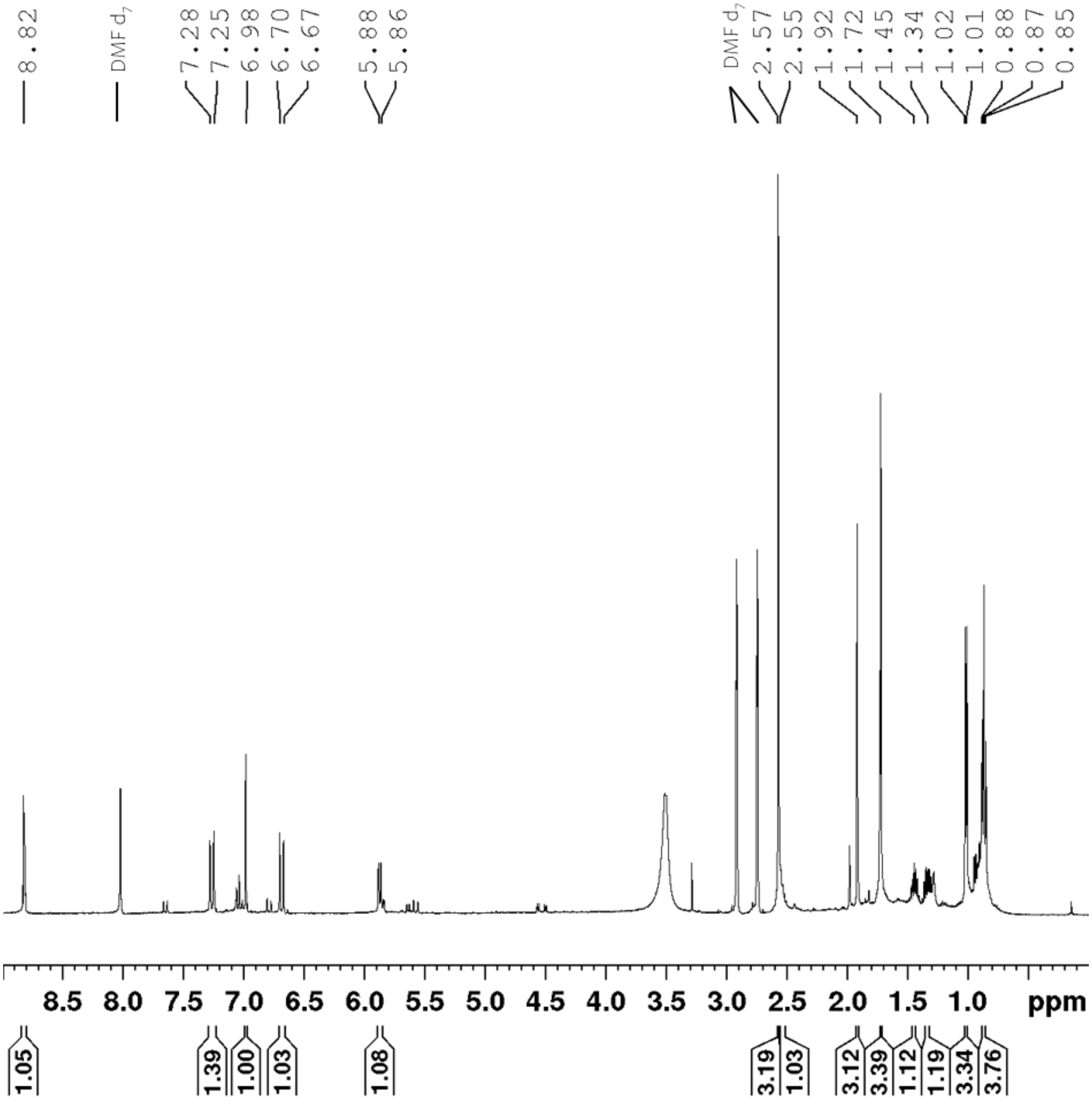

Figure S17: ^{13}C NMR spectrum (175 MHz, DMF-*d7*) of compound **22.**

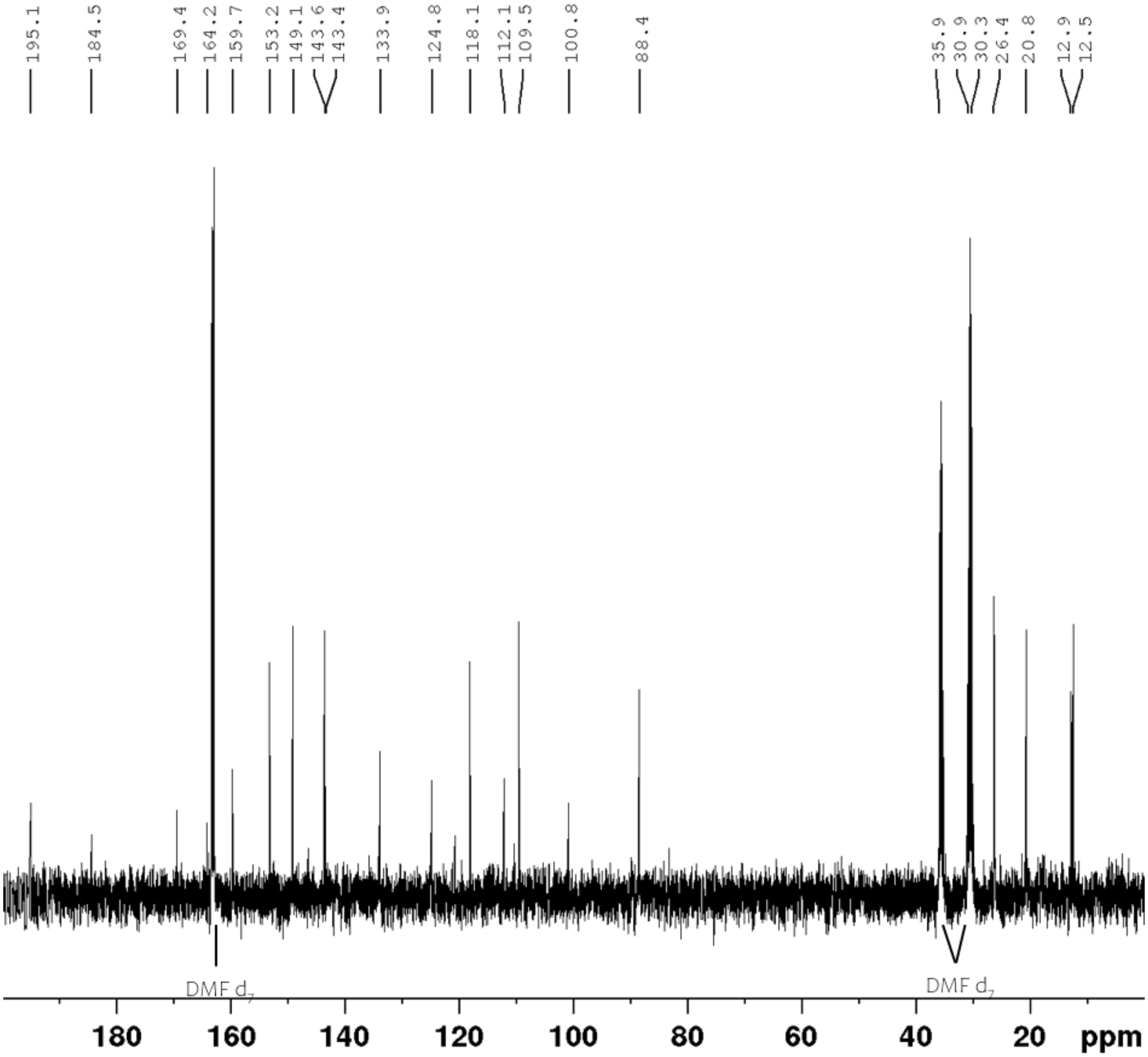

Figure S18: COSY NMR spectrum (700 MHz, DMF-*d7*) of compound **22.**

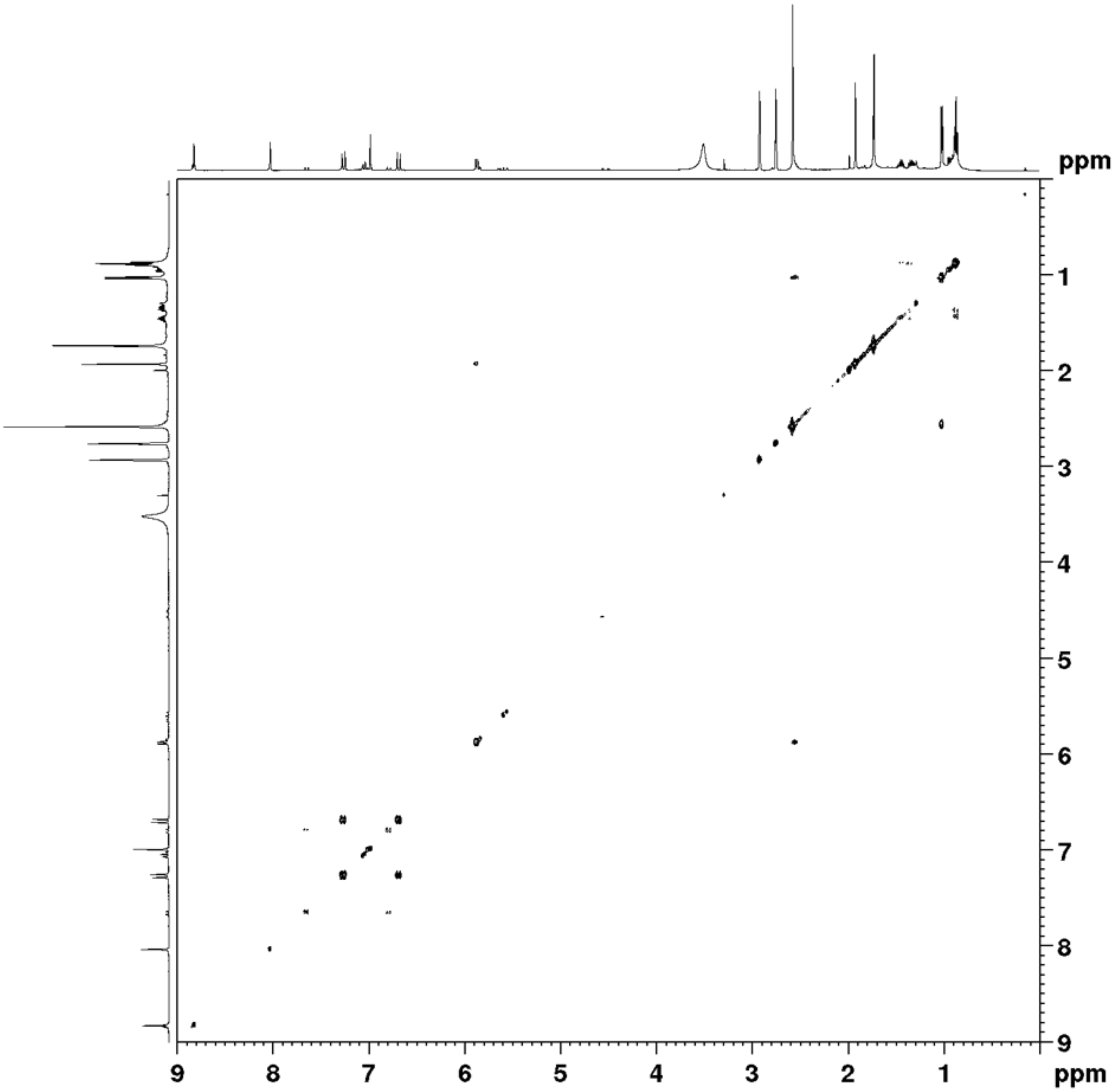

Figure S19: HSQC NMR spectrum (700 MHz, DMF-*d7*) of compound **22.**

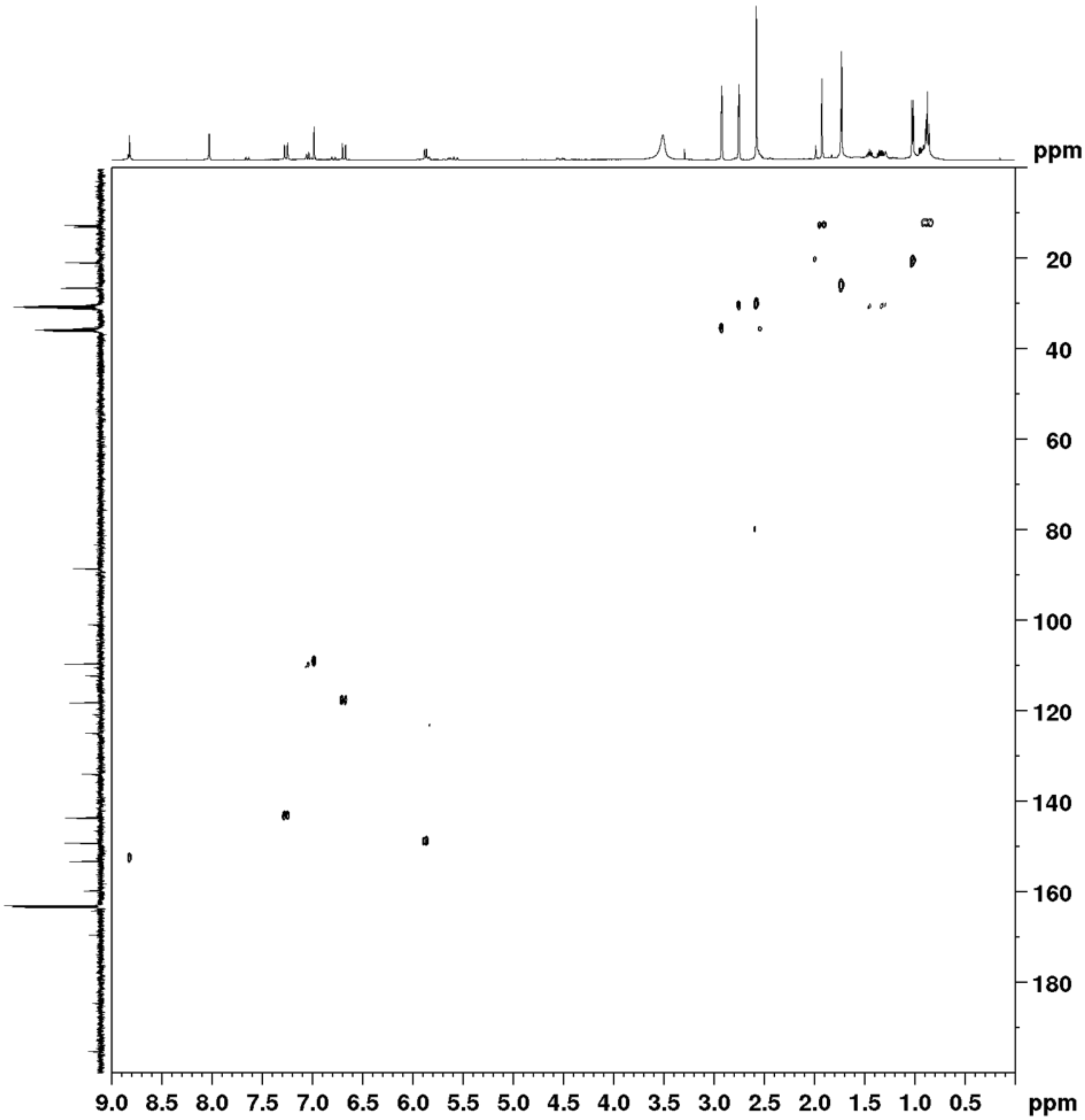

Figure S20: HMBC NMR spectrum (700 MHz, DMF-*d7*) of compound **22.**

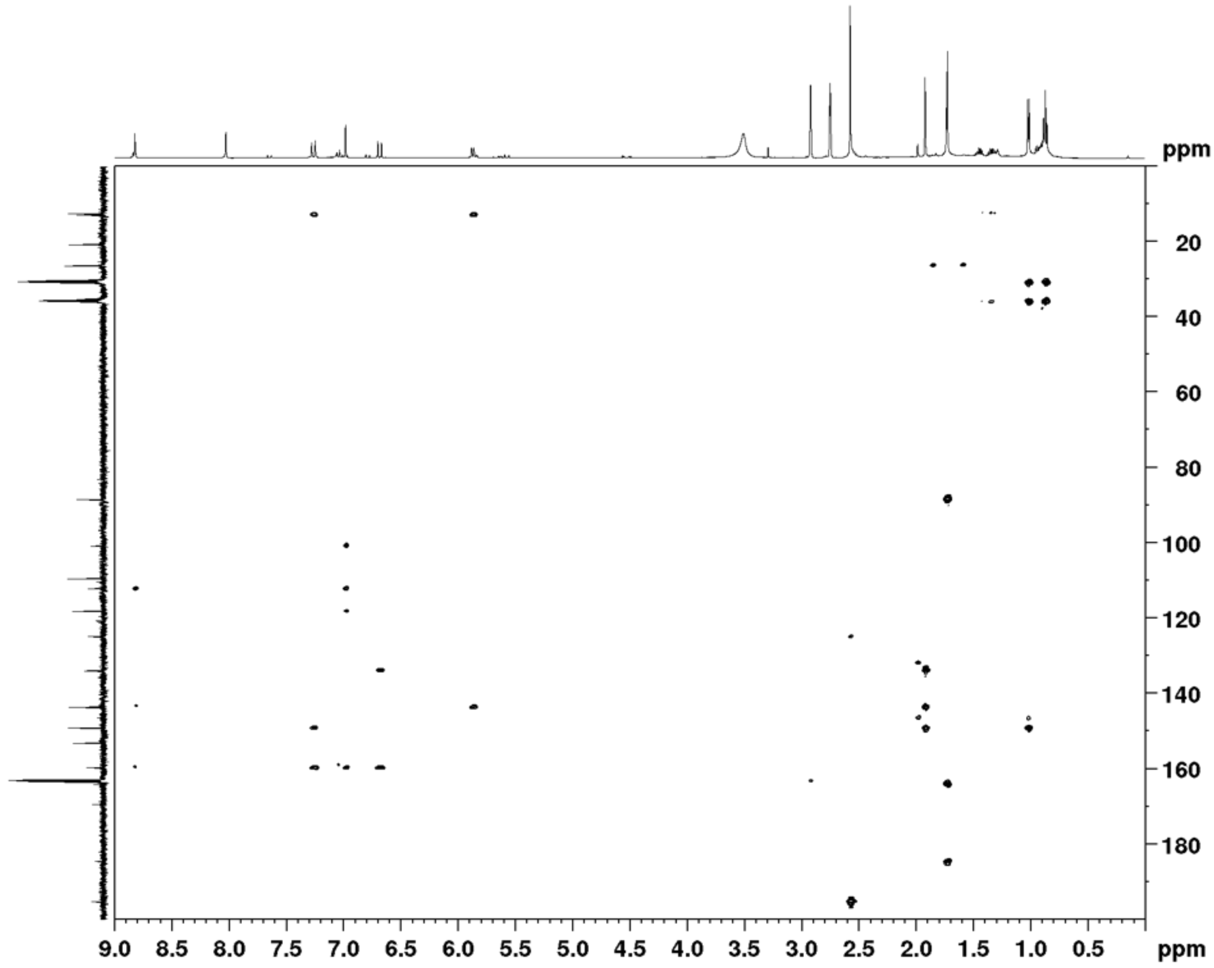

Figure S21: UV spectra in methanol of isolated azaphilone ***22***

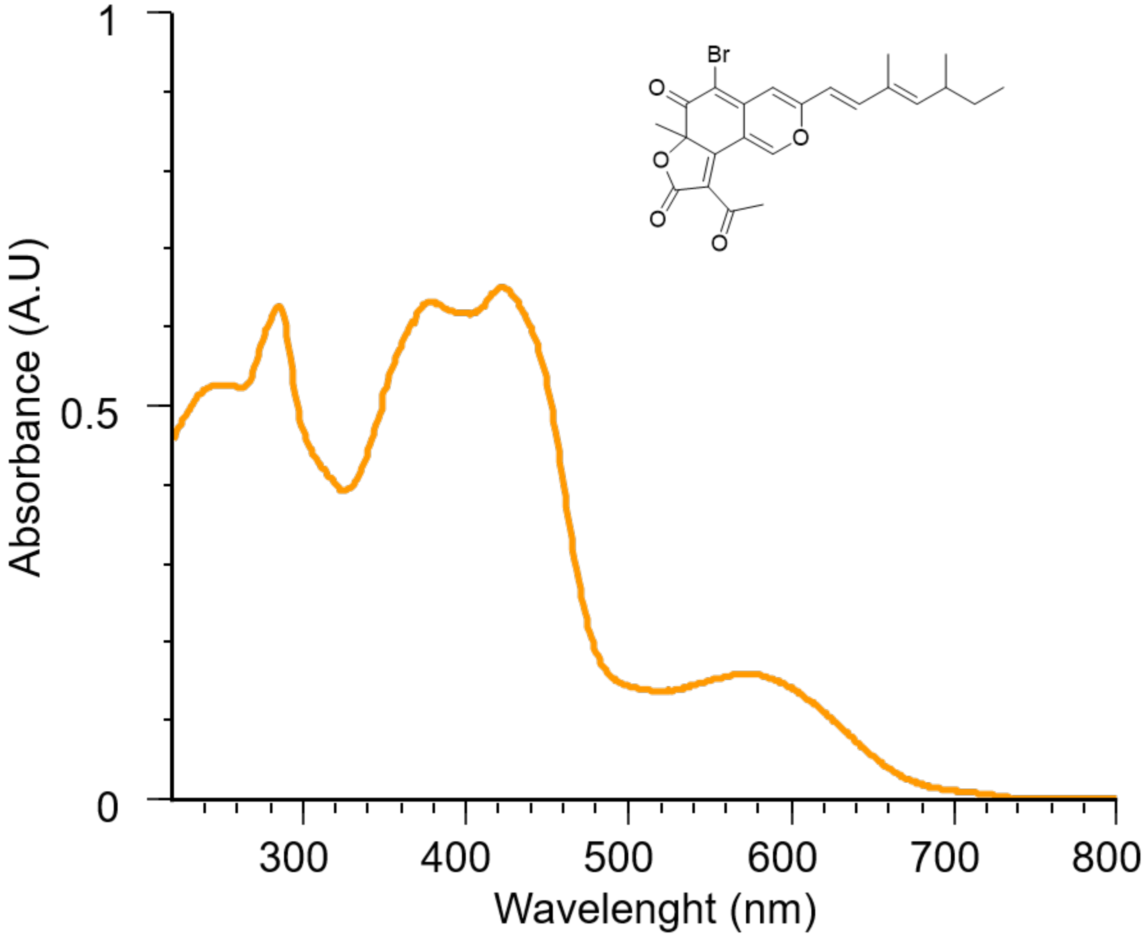

References

1. Hebra, T. *et al.* Dereplication, Annotation, and Characterization of 74 Potential Antimicrobial Metabolites from *Penicillium sclerotiorum* Using t-SNE Molecular Networks. *Metabolites,* **11**, 444 (2021).
2. Gao, J.-M., Yang, S.-X. & Qin, J.-C. Azaphilones: Chemistry and Biology. *Chem. Rev.,* **113**, 4755–4811 (2013).
3. Liu, Z. *et al.* Identification of anti-inflammatory polyketides from the coral-derived fungus *Penicillium sclerotiorun*: In vitro approaches and molecular-modeling. *Bioorg. Chem.,* **88**, 102973 (2019).
4. Son, S. *et al.* Structures and biological activities of azaphilones produced by Penicillium sp. KCB11A109 from a ginseng field. *Phytochemistry,* **122**, 154–164 (2016).
5. Arai, N. *et al.* Isochromophilones III -VI, Inhibitors of Acyl-CoA: Cholesterol Acyltransferase Produced by *Penicillium multicolor* FO-3216. *J. Antibiot.,* **48**, 696–702 (1995).
6. Aldridge, D. C. *et al.* Metabolites of *Nectria coccinea*. *J. Chem. Soc., 1* 2136 (1972) doi:10.1039/p19720002136.
7. Sorres, J., Sabri, A., Brel, O., Stien, D. & Eparvier, V. Ilicicolinic acids and ilicicolinal derivatives from the fungus *Neonectria discophora* SNB-CN63 isolated from the nest of the termite *Nasutitermes corniger* found in French Guiana show antimicrobial activity. *Phytochemistry,* **151**, 69–77 (2018).
8. Nirma, C., Eparvier, V. & Stien, D. Antibacterial Ilicicolinic Acids C and D and Ilicicolinal from *Neonectria discophora* SNB-CN63 Isolated from a Termite Nest. *J. Nat. Prod.,* **78**, 159–162 (2015).
9. Li, Y., Liu, D., Cen, S., Proksch, P. & Lin, W. Isoindolinone-type alkaloids from the sponge-derived fungus Stachybotrys chartarum. *Tetrahedron,* **70**, 7010–7015 (2014).
10. Takamatsu, S. *et al.* A Novel Testosterone 5.ALPHA.-Reductase Inhibitor, 8',9'-Dehydroascochlorin Produced by *Verticillium* sp. FO-2787. *Chem. Pharm. Bull.,* **42**, 953–956 (1994).
11. Mori, K. & Takechi, S. Synthesis of the natural enantiomers of ascochlorin, ascofuranone and ascofuranol. *Tetrahedron,* **41**, 3049–3062 (1985).
12. Huang, X., Zhang, W., Tang, S., Wei, S. & Lu, X. Collaborative Biosynthesis of a Class of Bioactive Azaphilones by Two Separate Gene Clusters Containing Four PKS/NRPSs with Transcriptional Crosstalk in Fungi. *Angew. Chem. Int. Ed.,* **59**, 4349–4353 (2020).
13. Matsuzaki, K., Tahara, H., Inokoshi, J., Tanaka, H., Masuma, R., Omura, S. New Brominated and Halogen-less Derivatives and Structure-activity Relationship of Azaphilones Inhibiting gp120-CD4 Binding. *J. Antibiot.*., **51,** 1004–1011 (1998)
14. Pairet, L., Wrigley, K., Chetland, I., Reynolds, E.E., Hayes, M.A., Holloway, J., Ainsworth, A.M., Katzer, W., Cheng, X-M., Hupe, D.J., Charlton, P., Doherty, A.M. New Brominated and Halogen-less Derivatives and Structure-activity Relationship of Azaphilones Inhibiting gp120-CD4 Binding. *J. Antibiot.*, **48,** 913–923 (1995)
